# Influence of breast cancer risk factors on proliferation and DNA damage in human breast glandular tissues: role of intracellular estrogen levels, oxidative stress and estrogen biotransformation

**DOI:** 10.1007/s00204-021-03198-7

**Published:** 2021-12-18

**Authors:** Juliane Wunder, Daniela Pemp, Alexander Cecil, Maryam Mahdiani, René Hauptstein, Katja Schmalbach, Leo N. Geppert, Katja Ickstadt, Harald L. Esch, Thomas Dandekar, Leane Lehmann

**Affiliations:** 1 Chair of Food Chemistry, Institute of Pharmacy and Food Chemistry, University of Würzburg, Am Hubland, 97074 Würzburg, Germany; 2 Chair of Bioinformatics, University of Würzburg, Würzburg, Germany; 3 Chair of Mathematical Statistics with Applications in Biometrics, TU Dortmund University, Dortmund, Germany

**Keywords:** Estrogens, Human breast, Multiple linear regression, Metabolic network model

## Abstract

Breast cancer etiology is associated with both proliferation and DNA damage induced by estrogens. Breast cancer risk factors (BCRF) such as body mass index (BMI), smoking, and intake of estrogen-active drugs were recently shown to influence intratissue estrogen levels. Thus, the aim of the present study was to investigate the influence of BCRF on estrogen-induced proliferation and DNA damage in 41 well-characterized breast glandular tissues derived from women without breast cancer. Influence of intramammary estrogen levels and BCRF on estrogen receptor (ESR) activation, ESR-related proliferation (indicated by levels of marker transcripts), oxidative stress (indicated by levels of *GCLC* transcript and oxidative derivatives of cholesterol), and levels of transcripts encoding enzymes involved in estrogen biotransformation was identified by multiple linear regression models. Metabolic fluxes to adducts of estrogens with DNA (E-DNA) were assessed by a metabolic network model (MNM) which was validated by comparison of calculated fluxes with data on methoxylated and glucuronidated estrogens determined by GC– and UHPLC–MS/MS. Intratissue estrogen levels significantly influenced ESR activation and fluxes to E-DNA within the MNM. Likewise, all BCRF directly and/or indirectly influenced ESR activation, proliferation, and key flux constraints influencing E-DNA (i.e., levels of estrogens, *CYP1B1*, *SULT1A1, SULT1A2*, and *GSTP1*). However, no unambiguous total effect of BCRF on proliferation became apparent. Furthermore, BMI was the only BCRF to indeed influence fluxes to E-DNA (via congruent adverse influence on levels of estrogens, *CYP1B1* and *SULT1A2*).

**Supplementary Information:**

The online version contains supplementary material available at 10.1007/s00204-021-03198-7.

## Introduction

The development of breast cancer is associated with an exposure to increased levels of circulating estrogens e.g., 17β-estradiol (E2), estrone (E1) and other endogenous steroids over a prolonged period of time (Endogenous Hormones Breast Cancer Collaborative Group 2002, 2013; Colditz and Bohlke 2014). Recently, it was shown that modifiable risk factors (obesity, smoking as well as intake of ethinyl-E2 and E2-releasing drugs) associated with both increased breast cancer risk (Grosse et al. 2009; Colditz and Bohlke 2014; Gaudet et al. 2017; Collaborative Group on Hormonal Factors in Breast Cancer 2019; Gram et al. 2019) and higher levels of circulating E2 and E1 (Endogenous Hormones Breast Cancer Collaborative Group 2003, 2011, 2013) also influenced estrogen levels in human breast glandular and adipose tissues (Pemp et al. 2020).

The current understanding of the molecular etiology of breast cancer hypothesizes biotransformation of E2 and E1 within the breast tissue to catechols and subsequent oxidation to genotoxic quinones resulting in DNA damage via formation of estrogen–DNA adducts or via formation of reactive oxygen species resulting in oxidative stress (Yager 2015).

**Fig. 1:**
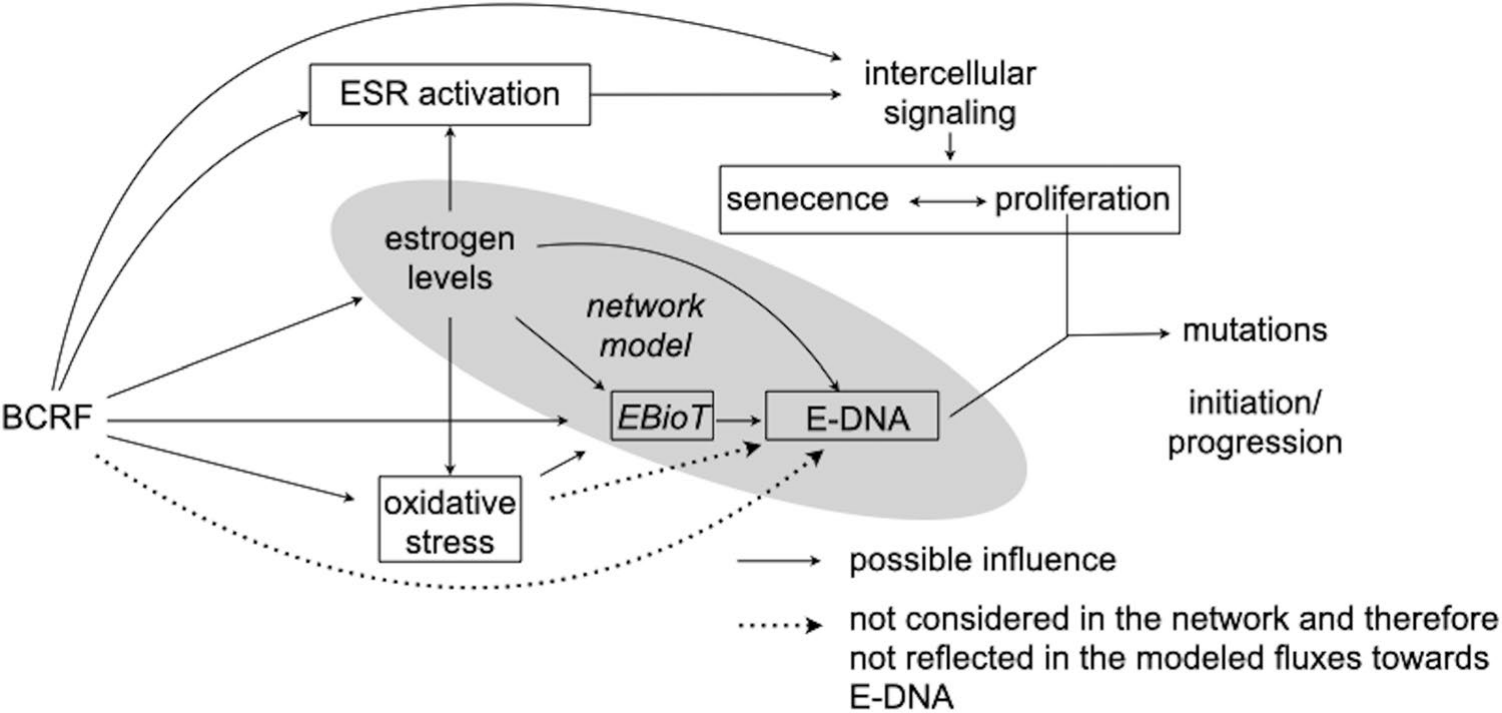
Possible ways of interaction of breast cancer risk factors (BCRF) with cell proliferation and estrogen biotransformation resulting in formation of adducts of estrogens with DNA (E-DNA) in human breast glandular tissue. Variables in boxes were investigated as dependent variables in “Multiple linear regression models”. *EBioT* enzymes involved in biotransformation of estrogens

In addition, estrogen receptor alpha (ESR1)-mediated stimulation of proliferation of the epithelial cells contributes to conversion of DNA damage into mutations (i.e., tumor initiation) as well as tumor promotion and progression (Yager 2015). ESR1-mediated proliferation involves complex intercellular signaling between epithelial and stromal cells (Lanigan et al. 2007). After activation of ESR1 and progesterone receptor (PGR), several downstream regulators for intercellular signaling, e.g., amphiregulin (AREG, McBryan et al. 2008), estrogen receptor beta (ESR2, Warner et al. 2020), GATA binding protein 3 (GATA3, Chou et al. 2010), transforming growth factor beta 1 (TGFB1, Massague 2012), trefoil factor 1 (TFF1, Amiry et al. 2009; Buache et al. 2011), Wnt family member 4 (WNT4, Alexander et al. 2012), and intracellular signaling, e.g., cyclin D1 (CCND1, Sicinski et al. 1995), cyclin-dependent kinase inhibitor 1A (CDKN1A, Kreis et al. 2019) and cyclin-dependent kinase inhibitor 1B (CDKN1B, Ding et al. 2019) maintain controlled proliferation of mammary epithelial cells (Lanigan et al. 2007). Thus, tumor formation (Yager 2015) seems to depend on intramammary levels of both reactive estrogen biotransformation products and free estrogens able to activate ESR1 (Fig. 1). Yet, the association of intramammary levels of E2 and E1 with the signaling leading to ESR1-dependent proliferation in human breast tissues in women without breast cancer is hitherto unexplored.

Equally uncertain is the association of intramammary levels of E2 and E1 with the formation of DNA adducts by reactive estrogens: although the association of circulating estrogen levels with breast cancer risk suggests that intraglandular estrogen levels might be associated with levels of DNA-reactive estrogen quinones, the entirety of the multitude of enzymes activating (cytochrome P450s, CYPs) and deactivating estrogens (UDP glucuronosyltransferases, UGTs, and sulfotransferases, SULTs), and its metabolites (catechol-*O*-methyltransferase COMT, NAD(P)H quinone dehydrogenase 1, NQO1, glutathione *S*-transferases GSTs; Online Resource 1) will determine to which extent high intraglandular estrogen levels will contribute to formation of adducts of estrogens with DNA. Moreover, it has been shown recently that levels of transcripts encoding enzymes in estrogen (biotrans)formation influence intramammary estrogen levels (Pemp et al. 2020), stressing the importance of considering intratissue biotransformation.

Besides the well-known effect of substrates and/or products on the enzyme kinetics and expression of the genes encoding enzymes catalyzing the biotransformation of estrogens, the expression is often regulated by ligand-dependent transcription factors which enables tissues to respond to endogenous and exogenous signals. For example, the expression of enzymes involved in biotransformation of estrogens is regulated by oxidative stress (Nebert et al. 2000; Zordoky and El-Kadi 2009; Dinkova-Kostova and Talalay 2010; Lu 2013; Fig. 1). Of note, in addition to elevated estrogen levels, oxidative stress generated by inflammation (Lonkar and Dedon 2011) is discussed to contribute to the positive association between obesity-related body mass index (BMI) and breast cancer risk (Himbert et al. 2017).

Since multiple (iso)enzymes compete for estrogens and vice versa (Online Resource 1)*,* the impact of intramammary levels of E2 and E1 on formation of adducts of estrogens with DNA can only be predicted by appropriate statistical or bioinformatical methods. Statistical methods such as linear regression are suitable to identify central elements in the metabolism, but they are not able to predict the impact of different estrogen and transcript level scenarios. In contrast, the response of the biological system can be predicted by investigating the dynamic behavior of metabolic processes by means of simulation (Rezola et al. 2015); e.g., using a constraint-based modeling based on a stoichiometric matrix (Cecil et al. 2011). The resulting fluxes describe the activity of the respective reaction in the whole network.

Thus, the aim of the present study was to investigate the influence of intramammary estrogen levels and breast cancer risk factors on ESR1-dependent intercellular signaling and bioactivation of E2 and E1 to genotoxins and formation of adducts of estrogens with DNA (Fig. 1).

To achieve his aim,

- the influence of breast cancer risk factors and estrogen levels on ESR activation and oxidative stress as well as the relevance thereof for cell proliferation was determined by multiple linear regression models.
- possible targets of breast cancer risk factors in estrogen biotransformation, sensitive to modulation by estrogen levels and oxidative stress were identified by multiple linear regression models.

To assess the relevance of these influences for the formation of adducts of estrogens with DNA,

- a network model comprehensively describing activation and deactivation reactions in estrogen biotransformation was developed and validated.
- the metabolic fluxes to adducts of estrogens with DNA were modeled using levels of E2 and E1 as well as levels of transcripts encoding enzymes involved in biotransformation of E2 and E1 determined previously in well-characterized human breast glandular tissues (Pemp et al. 2019, 2020) as flux constraints.
- the influence of breast cancer risk factors and of levels of biomarkers for oxidative stress on metabolic fluxes to adducts of estrogens with DNA were determined by multiple linear regression (Fig. 1).

## Materials and methods

### Origin of biospecimens

Breast tissue specimens were obtained from 47 adult women without breast cancer undergoing reduction mammoplasty between 2010 and 2015. All women participating in the study gave their written informed consent prior to their inclusion in the study. Women with a personal and/or family history of breast cancer were not eligible for participation. All participants were asked to complete a questionnaire about information on age, height, weight, parity (parous/nulliparous), smoking habits (never smoker, current smoker, current nonsmoker the latter two with daily cigarette consumption), intake of estrogen-active drugs as well as intake of dietary supplements containing phytoestrogens. Individual BMI was calculated in kg/m^2^. The study population consisted of 26 premenopausal, 12 perimenopausal and 9 postmenopausal women. However, the perimenopausal group may also contain pre- as well as postmenopausal women, since allocation to each group was based on the age of the participants (Pemp et al. 2019).

### Tissue characterization and preparation

Of the 47 biospecimens only 44 were eligible for the metabolic network model (for two biospecimens no levels of transcripts could be obtained and one biospecimen had to be excluded to achieve the validation of the metabolic network). In addition, two women did not volunteer information on the use of estrogen-active drugs and in one case no analysis of the oxidation products of cholesterol could be performed. Thus, only 41 biospecimen were eligible for multiple linear regression models.

All glandular and adipose tissues were prepared and characterized previously (Pemp et al. 2019, 2020). If tissue appearance did not allow to quickly generate aliquots of apparently plain adipose and glandular tissue with less than 15% adhering adipose tissue, the biospecimens were flash-frozen in liquid nitrogen and glandular tissue was isolated from cryosections (40 µm) at maximum − 20 °C using a scalpel. Tissues were stored at − 80 °C until further use. Characterization of biospecimen included their mass percentages of oil (oil%), percentage of area covered by intra- and interstromal adipocytes and lobule type and were reported already. Oil% in tissues was determined gravimetrically after extraction with chloroform. Percentage of area covered by intra- and interstromal adipocytes and lobule type of glandular tissues was estimated microscopically (Leica LMD6500) in cryosections (10 µm) of glandular tissues stained with hematoxylin and eosin Y by two different persons and coded slides according to Pemp et al. (2019, 2020).

### Instrumental analysis of E2, E1, 2-methoxy-E1, E1 sulfate, and E1 glucuronide

E2, E1 and 2-methoxy(MeO)-E1 were determined by GC–MS/MS (Varian 450-GC, 300-MS; Bruker Daltonics, Bremen, Germany) whereas E1 sulfate (E1-S) and E1 glucuronide (E1-G) were determined by LC–MS/MS (QTrap^®^ 5500; AB Sciex, Darmstadt, Germany). Tissue levels of E2, E1, 2-MeO-E1, E1-S were quantified using their respective deuterated derivatives and E1-G was monitored qualitatively. All data have already been published by Pemp et al. (2019, 2020). Data used in statistical analyses are presented in Online Resource 2.

### Determination of transcript levels

Analysis of transcript levels of genes encoding enzymes involved in E2 (biotrans)formation and regulation thereof was performed using customized Taqman^®^ Low Density Arrays and Taqman^®^ Gene Expression Assays as described by Pemp et al. (2019). Data used in statistical analyses are presented in Online Resource 3.

### Quantitation of intratissue levels of oxidation products of cholesterol

Analysis of the oxidation products of cholesterol (ChOL) formed by reactive oxygen species, i.e., 7β-hydroxy-ChOL, 5,6α-epoxy-ChOL and 5,6β-epoxy-ChOL (oxyChOLs), was performed using GC–MS/MS (Varian 450-GC, 300-MS; Agilent Technologies^®^ Deutschland GmbH, Böblingen, Germany) after extraction, separation from ChOL and derivatization with BSTFA using a deuterated internal standard for each analyte as described in Online Resource 4. Data used in statistical analyses are presented in Online Resource 5.

### Metabolic network model

Usually, all required pathways for the construction of the network are found in public data bases such as KEGG. However, only 14 of the 159 reactions needed to construct the network model were found in KEGG database. The remaining reactions were identified individually (Pemp et al. 2019). Reactions in estrogen biotransformation and isoenzymes considered are presented in Online Resource 1. Additionally, to model co-factor production, pathways of tricarbocylic acid cycle (or Krebs cycle), oxidative phosphorylation, pentose phosphate pathway and glycolysis were added to the estrogen biotransformation network. Methods, resources, and the pathways of the energy metabolism used were described in Cecil et al. (2011).

The network was set up using YANAsquare software (Schwarz et al. 2007). YANAsquare provides two opportunities to constrain the amount of metabolites and cofactors in the network: internal and external. When setting a metabolite as”internal” the production of it within the network is considered when calculating connected reactions. Thus, these metabolites/cofactors can be limited and influence the whole network (*S*-adenosylmethionine, glutathione, 3′-phosphoadenosine-5′-phosphosulfate, catechols, quinones). In contrast, an”external” metabolite/co-factor is available unlimitedly for the calculation of the fluxes (NADPH, UDPGA, sulfates, glucuronides, E1, E2).

For calculation of network fluxes (Online Resource 1), YANAsquare and a custom-made routine written in R were used (Cecil et al. 2011). As flux constraints (input data), levels of mRNA encoding enzymes (a) involved in estrogen biotransformation determined by TaqMan PCR (Online Resource 3) and (b) involved in energy metabolism determined by RNA sequencing (Rowley et al. 2011) were used. If levels of mRNA encoding enzymes involved in estrogen biotransformation were below LOD/LOQ, LOD/LOQ was used as flux constraint.

The calculation method was convex basis based: the first constraint represented the steady-state condition, i.e., no accumulation or depletion of the metabolites inside the network. The more reactions a metabolite is involved in, the more active is the synthesis reaction. The second constraint was the thermodynamic feasibility, which restricts some fluxes to being non-negative, because of their associated Gibbs free energy. Together with the input data, the model calculates the resulting fluxes within the network based on a third constraint, the non-decomposability condition, which ensures that the calculated solution comprises a minimal number of active reactions at steady state. This implies that these solutions cannot be decomposed into any smaller flux distributions without violating the steady-state constraint (summarized in Rezola et al. 2015). The resulting fluxes describe the activity of the respective reaction in the whole network even for reactions where input data has been available.

The fluxes calculated with data of the 44 women (Online Resource 6) were compared using statistical methods with levels of 2-MeO-E1 and E1-G determined qualitatively and quantitatively in breast tissues (Online Resource 2): Median metabolic fluxes to E1-G and 2-MeO-E1 were compared with the detection of E1-G and 2-MeO-E1, respectively < or > LOD by unpaired Wilcoxon test. Correlation of metabolic fluxes with levels of 2-MeO-E1 and 2-MeO-E1 > LOD were analyzed by Spearman’s rank correlation. The levels of 4-MeO-E1 and 2/4-MeO-E2 were < LOD in all samples, whereas levels of 2-MeO-E1 (with a comparable LOD; Pemp et al. 2020) were quantifiable in some samples (Pemp et al. 2020). Thus, the differences of the metabolic fluxes to 2-MeO-E1 and to the other methoxylated estrogens were compared with “0” by Friedmann test followed by Dunn´s post hoc test.

### Statistical methods

All statistical analyses were performed with the statistical programming language R, version 3.5.2 (R Core Team 2020) and all tests of statistical significance were two sided. Whenever multiple comparisons were performed, *P* values were adjusted using Holm's method.

#### Metabolic network validation

Differences between two and more than two data sets were investigated using unpaired Wilcoxon und Friedmann test (Dunn’s post hoc test), respectively. Spearman’s rank correlation was used to analyze correlations between data.

#### Principal component analysis

Number of principle components (PC), which were calculated in R for specific groups of explanatory variables (exVARs), was chosen using the scree plots according to the elbow criteria and all PC considered explained at least two third of the variation of the original variables.

#### Analyses of independence of variables

Spearman’s rank correlation analysis was performed to identify collinearity between numerical exVARs which might hinder each other’s selection and/or influence each other’s *P* values within the models. In the case of variables with > 1 level below LOD or LOQ, correlation was calculated with randomly distributed ranks for ties 10,000 times and highest Spearman correlation coefficients and lowest *P* values were used to rather overestimate collinearity. Relationship between categorical and numerical exVARs was evaluated by comparison of medians using unpaired Wilcoxon tests. Indications for relationships between variables and possible consequences for the selection of exVARs are given for each model in Online Resource 7.

#### Multiple linear regression models

To test the association of every possible exVAR with the dependent variable, the variable explaining the dependent variable best is chosen by an automatic procedure. Subsequently, all possible exVARs are added one after another to the first one, ultimately choosing the one improving the model most, applying the Akaike information criterion. This is repeated until the model cannot be further improved by adding exVARs. Thus, each exVAR selected into the model contributes to modeling the dependent variable. Significance of the association is expressed by *P* values and magnitude of impact is expressed by coefficients of regression. The choice of exVARs is discussed in the results section and more detailed information is given in Online Resource 7. If in the computed model observations with Cook’s distance > 1 appeared, they were removed, and the model was computed anew. This process was repeated until no conspicuous observations occurred. To achieve normal distribution, dependent variables were logarithmized. Data distributions were evaluated in Quantile–Quantile plots with simulated confidence bands. Constant standard deviations of the errors were evaluated using scale-location plots. To check the model assumption of independent identically distributed errors, the residual vs. fitted values plot was used.

The adjusted coefficients of determination, the numbers of conspicuous observations removed, the numbers of observations contributing to the final models (maximum of 41 because of two specimens without information on the intake of estrogen-active drugs and one specimen without information on the intake of oxyChOLs).

The ratio of observations per exVAR of each final model was also given and to achieve accurate estimation of regression coefficients, at least two observations per exVAR (Austin and Steyerberg 2015) were aimed for.

In addition, the regression coefficients (which represent the mean changes in the dependent variables for one unit of change in the respective exVAR while holding other predictors in the models constant), their confidence interval, as well as the *P* values of each exVAR selected are given in Online Resource 7.

## Results

Breast cancer risk factors such as BMI, smoking and intake of estrogen-active drugs but also oil% influenced estrogen levels in glandular tissues (Pemp et al. 2020). To investigate the relevance of this observation, the influence of intraglandular estrogen levels and breast cancer risk factors on ESR1-mediated cell proliferation and formation of adducts of estrogens with DNA was investigated by multiple linear regression models and metabolic network modeling (Fig. 1).

### Influence of intratissue estrogen levels and of variables associated with lifestyle on activation of ESR1

Proliferation of breast epithelial cells is a result of complex intercellular signaling between epithelial and stromal cells which requires activation of ESR1 (Lanigan et al. 2007). Activated ESR1 is able to bind to the estrogen response element in the promoter region of genes encoding GATA3, AREG and TFF1, which are involved in intercellular signaling regulating epithelial cell proliferation (Eeckhoute et al. 2007; McBryan et al. 2008; Amiry et al. 2009). Also, the induction of the expression of the gene encoding PGR represents a well-characterized marker for activation of ESR1, although the PGR gene does not exhibit an estrogen response element in its regulatory sequences (Thomas and Gustafsson 2015). Furthermore, E2-stimulated proliferation of the epithelial cells is mediated by ligands for membrane bound receptors, e.g., WNT4 (stimulation) as well as e.g., TGFB1 (inhibition) even though regulation of expression of WNT4 and TGFB1 via estrogen response elements is not known.

Thus, to investigate the activation of ESR1 in glandular tissues, the following dependent variables and potential exVARs were identified:

1. Dependent variables: because their expression is known to be controlled by activated ESR1, levels of *AREG, GATA3* and *TFF1* (estrogen response element dependent) as well as *PGR, TGFB1*, *WNT4* (estrogen response element independent) were chosen as dependent variables.
2. Possible exVARs:
3. Since intracellular levels of E2, E1 and E1-S in glandular tissues correlated with each other (Online Resource 7), they were combined by principal component analysis to avoid collinearity. PC_E_1 of estrogen levels in glandular tissues was characterized by comparable strong positive contributions of E2 and E1, as well as (smaller) contribution of E1-S and explained 73% of the variance (Online Resource 2). PC_E_2 essentially differentiated E2 and E1 exhibiting similar negative values, from E1-S (positive) explaining 21% of the variance, whereas PC_E_3 differentiated E2 (positive) from E1 (negative) explaining 6% of the variance. Thus, PC_E_1 was chosen as exVAR to represent intracellular levels of estrogens able to activate ESR1.
4. If breast cancer risk factors exert an effect on ESR1 activation by influencing intracellular estrogen levels, the exVAR PC_E_1 will account for this effect. However, to account for possible other relations between breast cancer risk factors and activation of ESR1 (e.g., indirectly via activation of other signaling pathways) as well, breast cancer risk factors (BMI, smoking, intake of estrogen-active drugs) were additionally considered.
5. Transcript levels of *ESR1* and *ESR2* were considered as well.
6. Tissue characteristics oil% and lobule type were considered as well. Oil% in glandular tissues is not related to the BMI (Pemp et al. 2020) but to the presence of adipocytes in glandular tissues (Pemp et al. 2019), which might be a result of estrogen signaling (Gao and Dahlman-Wright 2013).
7. To account for nutritional xenoestrogens which might interact with the action of endogenous estrogens intake of dietary supplements containing phytoestrogens was included.

**Fig. 2:**
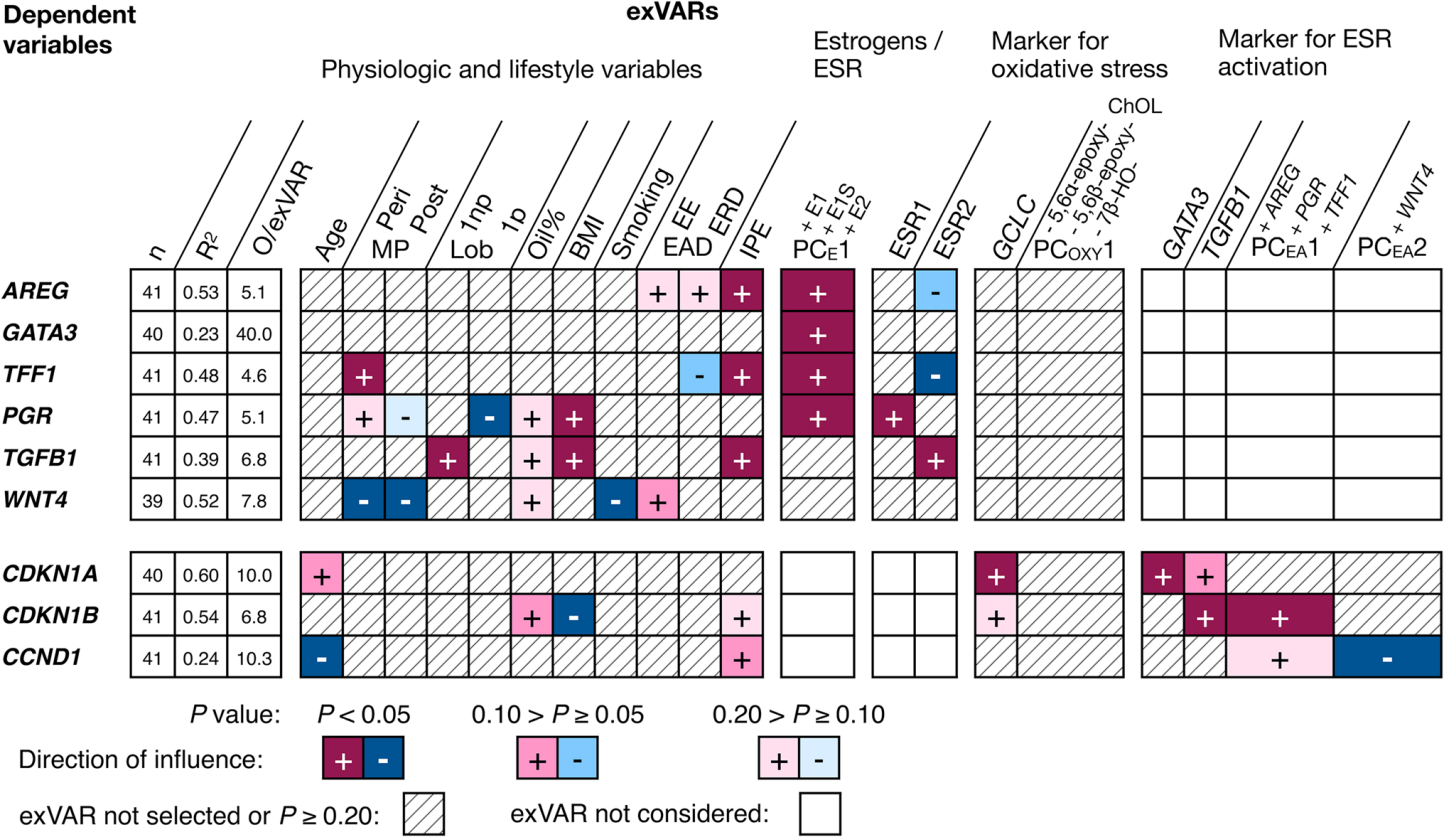
Influence of various exVARs on levels of transcripts of marker for ESR1 activation and proliferation identified by multiple linear regression models using stepwise forward selection as detailed in Online Resource 7. For each model, the number (*n*) of observations (O) contributing to the final model, the adjusted coefficient of determination (*R*^2^), and the observations (i.e., biospecimens) to exVAR ratio (O/exVAR) after forward selection of variables is given. *EAD* estrogen-active drug, *EE* ethinyl-E2, *ERD* E2-releasing drugs, *IPE* intake of dietary supplements containing phytoestrogens, *Lob 1np* lobule type 1 of nulliparous women, *Lob 1p* lobule type 1 of parous women, *periMP* perimenopausal, *postMP* postmenopausal

Levels of the marker transcripts *AREG, GATA3, TFF1* and *PGR* were positively influenced by PC_E_1 (*P* < 0.05, Fig. 2) indicating the direct quantitative role of intratissue estrogen levels on ESR1 activation in the glandular tissue and stressing the relevance of the increase in intratissue estrogens levels associated with breast cancer risk factors. Despite selection of the exVAR PC_E_1, perimenopausal status also influenced levels of *TFF1* positively (*P* < 0.05).

Furthermore, levels of both *AREG* and *TFF1* were positively influenced by intake of dietary supplements containing phytoestrogens (*P* < 0.05) suggesting an additional role of nutritional phytoestrogens in ESR1 activation in the glandular tissue. Furthermore, levels of *ESR2* influenced levels of *AREG* (0.10 > *P* ≥ 0.05) and *TFF1* (*P* < 0.05) negatively.

In contrast, levels of *PGR* (estrogen response element independent, Thomas and Gustafsson 2015) were positively influenced by levels of *ESR1* and by BMI (*P* < 0.05) suggesting further mechanisms influenced by BMI besides increasing estrogen levels (e.g., increased intramammary inflammation; Hardy et al. 2008; Iyengar et al. 2016).

Levels of the transcripts encoding the stromal signaling molecules TGFB1 and WNT4 were not influenced by PC_E_1; yet positively (*P* < 0.05) by intake of dietary supplements containing phytoestrogens, levels of *ESR2*, lobule type and BMI (*TGFB1* only) and negatively (*P* < 0.05) by smoking and peri- and postmenopause (*WNT4* only). Based on the role of TGFB1 (inhibition of proliferation) and WNT4 (induction of proliferation) in non-cancer tissues (Alexander et al. 2012; Massague 2012), these influences could result in less proliferation of breast epithelium in women without breast cancer.

#### Influence of variables associated with breast cancer risk factors, estrogen receptor activation and intercellular signaling on proliferation

1. Dependent variables: key regulators of the cell cycle are cyclin-dependent kinases which are activated by binding cyclins and inhibited by inhibitor proteins. Growth inhibitory signals, including DNA damage; induce expression of inhibitor proteins, e.g., p21 and p27 encoded by *CDKN1A* and *CDKN1B*, respectively, which inhibit all cyclin-dependent kinases, especially those regulating entry and progression through the S phase. However, this inhibition is overcome by the increased activity of cyclin D1-dependent kinases (Otto and Sicinski 2017). Mitogenic signals increase expression of cyclin D1 (encoded by *CCND1*) which ultimately causes senescent cells to enter the G1 phase. Thus, levels of *CCND1* (stimulation of proliferation) as well as *CDKN1A* and *CDKN1B* (inhibition of proliferation) were chosen as marker transcripts.
2. Putative exVARs: Levels of the transcripts known to be regulated directly or indirectly by ESR1 (serving as dependent variables in the previous section) reflect ESR activation in glandular tissues. To avoid collinearity, levels of *AREG*, *TFF1, PGR* and *WNT4* were combined by principal component analysis, resulting in PC_EA_1 (characterized by *AREG, TFF1* and *PGR*) and PC_EA_2 (characterized by *WNT4,* Online Resource 3). Levels of *GATA3* and *TGFB1* were directly included as potential exVAR.
  Since only levels of a few exemplary transcripts encoding proteins involved in intercellular communication were available to account for breast cancer risk factors influencing transcripts or proteins not determined in the present study, breast cancer risk factors were considered as exVARs as well (Fig. 1).

Unexpectedly, no exVAR tested influenced levels of *CCND1* positively at *P* < 0.05 (Fig. 2). Negative influence on levels of *CCND1* (*P* < 0.05) was observed for the exVARs age and PC_EA_2 (the latter is characterized by levels of *WNT4*).

In contrast, levels of *CDKN1A* known to be induced by DNA damage (Kreis et al. 2019) were positively (*P* < 0.05) influenced by levels of *GATA3* and *GCLC*, the latter being a marker transcript for oxidative stress. Furthermore, age and levels of *TGFB1* known to inhibit growth factor induced proliferation of epithelial cells contributed borderline positively (0.10 > *P* ≥ 0.05) to *CDKN1A* levels.

Furthermore, BMI contributed negatively (*P* < 0.05) to levels of *CDKN1B*, suggesting stimulation of proliferation. In addition, levels of *CDKN1B* were positively influenced by PC_EA_1 and levels of *TGFB1* (all *P* < 0.05).

Taken together, BMI was the only breast cancer risk factor influencing cell proliferation at different levels: BMI did not only influence estrogen levels (Pemp et al. 2020) which in turn influenced activation of ESR1 but also intracellular signaling by influencing levels of *TGFB1* which—in line with the molecular role of TGFB1 in intracellular signaling of non-cancer cells—in turn influenced markers for senescent cells positively. However, BMI also directly influenced one marker of senescent cells negatively (*P* < 0.05, Fig. 2). Yet, these influences were not reflected by the chosen marker for cell proliferation.

### Influence of breast cancer risk factors on (de)activation of E2 and E1

It has already been shown that intramammary levels of estrogens are influenced by breast cancer risk factors (Pemp et al. 2020). To assess whether the expression of genes encoding enzymes involved in estrogen biotransformation is influenced by breast cancer risk factors as well, the influence of breast cancer risk factors on levels of transcripts involved in estrogen biotransformation directly and indirectly via induction of oxidative stress was investigated using multiple linear regression models.

To investigate the relevance of these influences on the (de)activation of E2 and E1, fluxes to adducts of estrogens with DNA were modeled (“Identification of reactions within the metabolic network model influencing fluxes to adducts of estrogens with DNA”) and influences thereon were identified by multiple linear regression models (“Influence of breast cancer risk factors on metabolic fluxes to adducts of estrogens with DNA”).

### Influence of breast cancer risk factors and (oxidative) cellular stress on levels of transcripts encoding enzymes involved in biotransformation of E2 and E1

In general, expression of genes involved in biotransformation can be regulated by signaling pathways activated e.g., by oxidative stress (Zordoky and El-Kadi 2009), ESR1 (Hewitt et al. 2010; Ochsner et al. 2019) or substrates or products of the encoded enzymes.

**Fig. 3:**
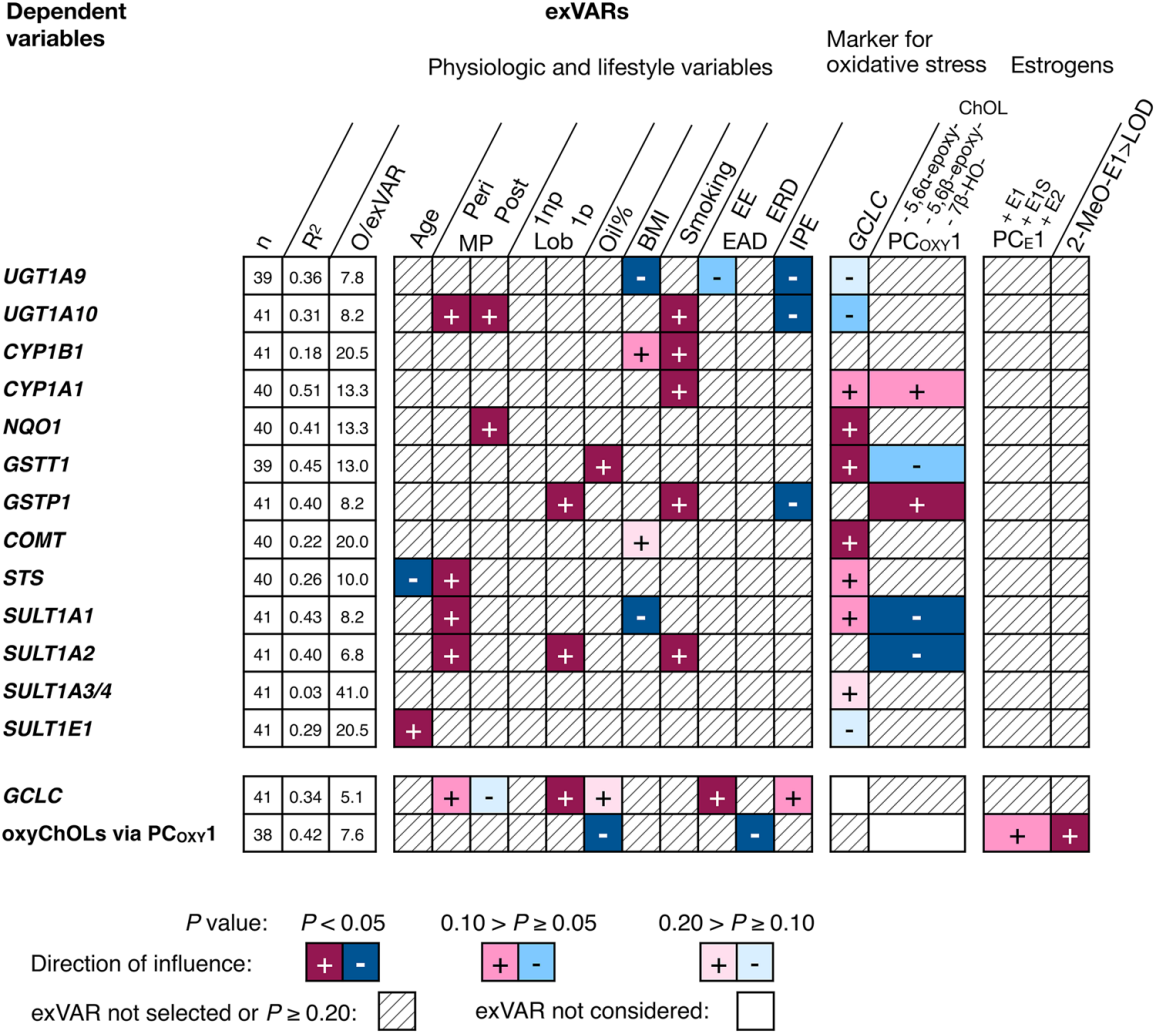
Influence of various exVARs on levels of transcripts encoding enzymes in biotransformation of E2 and E1, markers for (oxidative) cellular stress identified by multiple linear regression models using stepwise forward selection as detailed in Online Resource 7. For each model, the number (*n*) of observations (O) contributing to the final model, the adjusted coefficient of determination (*R*^2^), and the observations (i.e., biospecimens) to exVAR ratio (O/exVAR) after forward selection of variables is given. *EAD* estrogen-active drug, *EE* ethinyl-E2, *ERD* E2-releasing drugs, *IPE* intake of dietary supplements containing phytoestrogens, *Lob 1np* lobule type 1 of nulliparous women, *Lob 1p* lobule type 1 of parous women, *periMP* perimenopausal, *postMP* postmenopausal

Thus, exVARs considered were markers for short-term and long-term oxidative stress and levels of estrogens and activation of ESR1 in addition to breast cancer risk factors which were included to accounts for the action of breast cancer risk factors via mechanisms not reflected by the other exVARs. Since PC_E_1 and PC_EA_1 correlated significantly, to avoid collinearity, only PC_E_1 which encompassed more possible mechanisms than mere activation of ESR1 was chosen as putative exVAR (Fig. 3 and Online Resource 7).

PC_E_1 was not selected in any multiple linear regression model describing levels of transcripts encoding enzymes involved in estrogen biotransformation. Yet interestingly, intake of dietary supplements containing phytoestrogens influenced negatively (*P* < 0.05) levels of *UGT1A9, UGT1A10* and *GSTP1*. *UGT1A9* was also borderline negatively influenced by intake of estrogen-active drugs (0.10 > *P* ≥ 0.05).

BMI influenced borderline positively (0.10 > *P* ≥ 0.05) levels of *CYP1B1* as well as negatively levels of *SULT1A1* and *UGT1A9* (*P* < 0.05, Fig. 3), suggesting an increase in activation and decrease in inactivation of E1, E2 and and/or metabolites.

Smoking positively (*P* < 0.05) influenced levels of transcripts encoding enzymes involved in both activation (*CYP1A1* and *CYP1B1*) and deactivation (*GSTP1*, *SULT1A2, UGT1A10*) of pre-genotoxins in both potentially adverse and beneficial ways. Thus, its impact on activation of pre-genotoxins in the human breast will depend on the specific (pre-)genotoxin and profile of (iso)enzymes involved in its biotransformation.

Short-time (oxidative) cellular stress indicated by levels of *GCLC* influenced positively levels of *NQO1*, *GSTT1*, and *COMT* (all *P* < 0.05) and borderline positively (0.10 > *P* ≥ 0.05) levels of *CYP1A1, STS* and *SULT1A2*. PC_OXY_1 indicating long-term oxidative stress (Iuliano 2011) influenced levels of *SULT1A1* and *SULT1A2* (*P* < 0.05) as well as levels of *GSTT1* (0.10 > *P* ≥ 0.05) in a way indicating a positive or borderline positive association, respectively (negative influence of negative values). In contrast, levels of *GSTP1* (*P* < 0.05) and *CYP1A1* (*P* < 0.05) were influenced by PC_OXY_1 in a way indicating a (borderline) negative association (Fig. 3).

Thus, markers for short-term and long-term oxidative stress were not selected significantly together yet did not contradict each other either.

If breast cancer risk factors influenced levels of transcripts by modulation of the intracellular level of oxidative stress, selection of the exVARs PC_OXY_1 and *GCLC* might prevent selection of breast cancer risk factors into the previous multiple linear regression models (e.g., describing *NQO*, *GSTT1*, *COMT*, *SULT1A2*, *GSTP1*). Thus, to investigate if breast cancer risk factors influence (oxidative) cellular stress, levels of *GCLC* and PC_OXY_1 were investigated as dependent variable as well (Fig. 3).

### Influence of breast cancer risk factors on (oxidative) cellular stress

BMI or smoking did not significantly influence levels of *GCLC* or PC_OXY_1 indicating short-time and long-term oxidative stress, respectively (Fig. 3). Levels of PC_OXY_1 were negatively (*P* < 0.05) influenced by oil% and intake of E2-releasing drugs whereas levels of 2-MeO-E1 > LOD (*P* < 0.05) and PC_E_1 (0.10 > *P* ≥ 0.05) influenced levels of PC_OXY_1 positively.

Levels of *GCLC* were influenced positively (*P* < 0.05) by intake of ethinyl-E2 and lobule-type characteristic for parous women after age-related regression as well as intake of dietary supplements containing phytoestrogens (0.10 > *P* ≥ 0.05).

#### Metabolic network model and validation

To check the plausibility of the model (Online Resource 1), flux constraints of all reactions were set to “1”. The calculation of the model, however, failed due to unachievable constraints. Thus, to have more possibilities for a constraint conform solution of the calculation, minimum one reaction needed to be set to “0”. Since *UGT1A3/4* was not expressed in any specimen analyzed, its value was set to “0”. Since the biotransformation reactions of E2 and E1 are similar, similar flux values of the same enzyme were expected for E1 and E2 and indeed only fluxes of three out of 159 reactions differed (1.0 vs. 1.3, 0.1 vs. 0.2, and 1.0 vs. 1.3, Online Resource 8).

**Fig. 4:**
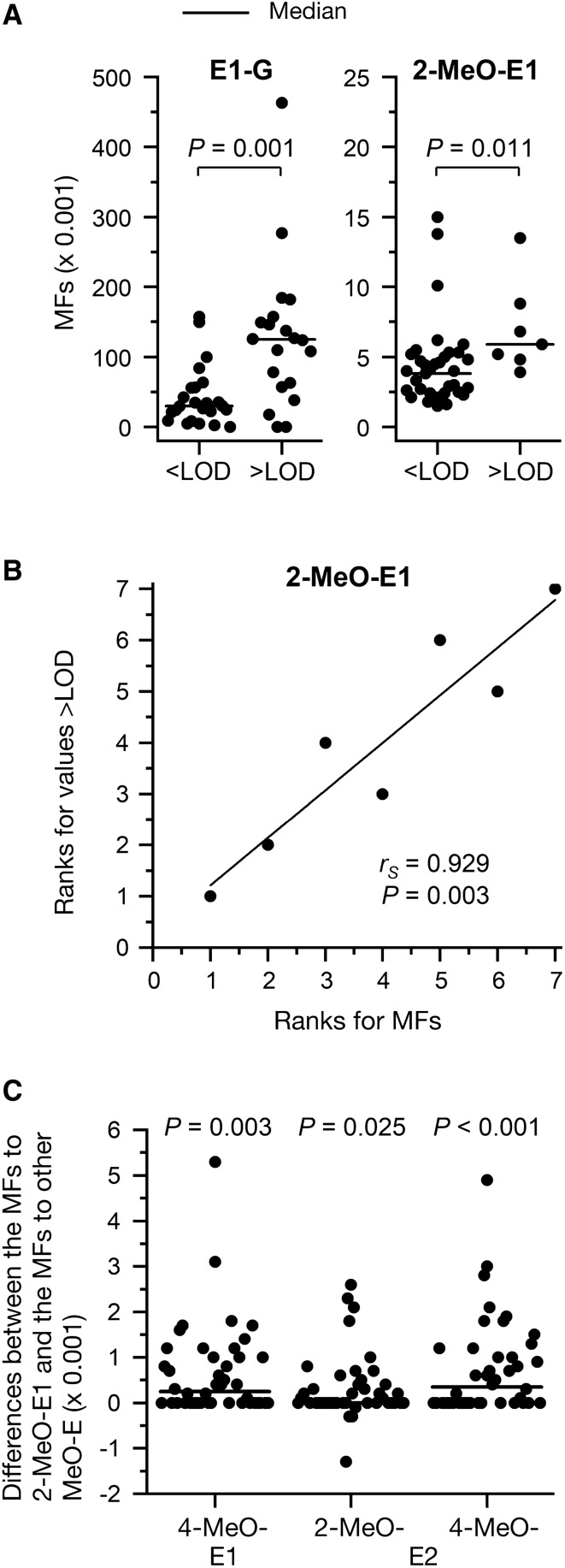
Validation of the metabolic network model. **A** Median modeled metabolic fluxes (MFs) to 2-MeO-E1 and E1-G were compared between samples with E1-G and 2-MeO-E1 levels below and above LOD by unpaired Wilcoxon test. **B** For samples exhibiting levels of 2-MeO-E1 above LOD, correlation between MFs to 2-MeO-E1 and levels of 2-MeO-E1 was analyzed by Spearman’s rank correlation analysis. Furthermore, individual differences in the MFs to the methoxylated metabolite detected at levels above LOD (2-MeO-E1) and of the methoxylated estrogens detected below LOD (MeO-E, i.e., 4-MeO-E1, 2-MeO-E2 and 4-MeO-E2) were analyzed by Friedman test (*P* = 0.001). Differences from “0” were identified by Dunn’s post hoc test (**C**). One difference between the MFs to 2-MeO-E1 and 2-MeO-E2 and one difference between the MFs to 2-MeO-E1 and 4-MeO-E2, respectively (both 11 × 0.001), are not shown

Subsequently, the metabolic network was modeled using levels of E2 and E1 as well as transcript levels derived from 44 breast glandular tissues as flux constraints. To validate the metabolic network model, levels and frequencies of detection above LOD of estrogen biotransformation products determined previously by GC– and UHPLC–MS/MS (Pemp et al. 2019) were compared statistically with fluxes towards the respective metabolites: fluxes to E1-G and 2-MeO-E1 were significantly lower in tissues exhibiting levels of E1-G and 2-MeO-E1 < LOD (Fig. 4). Furthermore, levels of 2 MeO-E1 > LOD correlated significantly with metabolic fluxes to 2-MeO-E1. Moreover, individual metabolic fluxes to 2-MeO-E1 (detectable > LOD in some samples) were higher than the fluxes to 4-MeO-E1, 2-MeO-E2, and 4-MeO-E2 (not detectable above the respective LODs), resulting in (a) positive values of the individual differences between fluxes to 2-MeO-E1 and the other methoxylated estrogens which were (b) significantly different from “0” (Fig. 4). Thus, the metabolic network was considered suitable to model fluxes to adducts of estrogens with DNA.

#### Identification of reactions within the metabolic network model influencing fluxes to adducts of estrogens with DNA

**Fig. 5:**
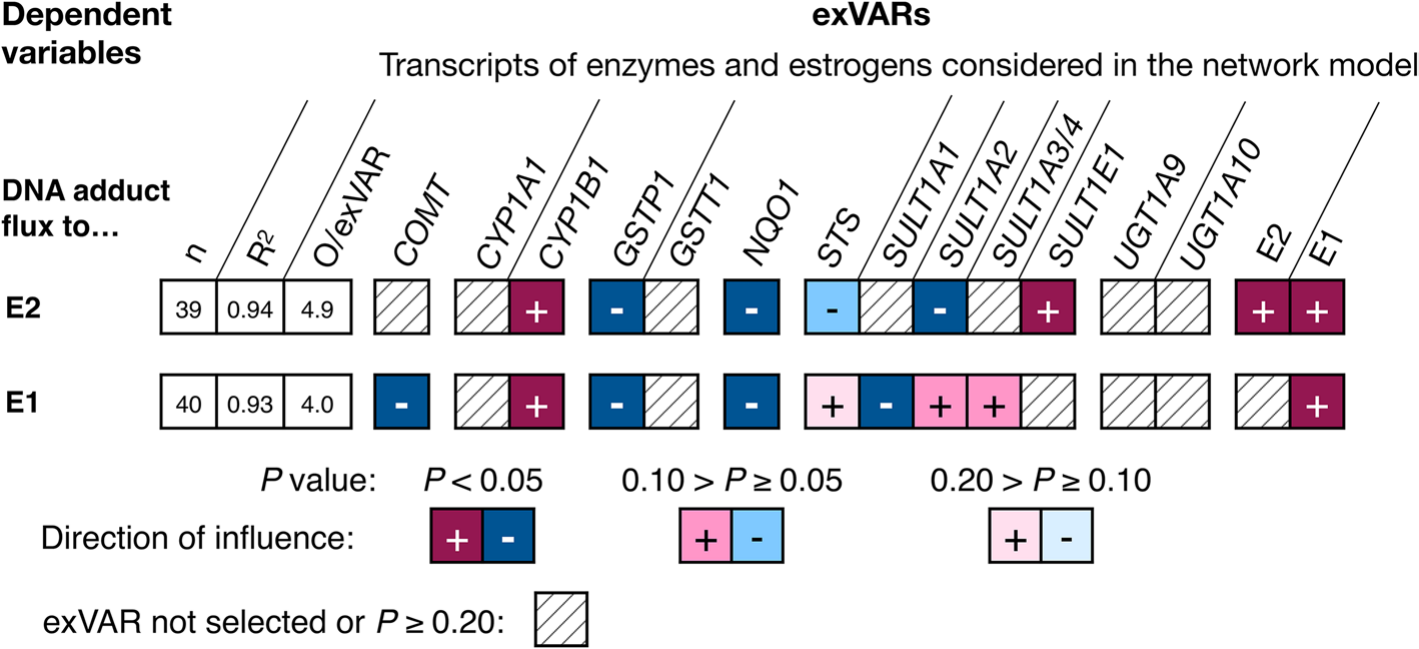
Influence of various exVARs on levels of calculated fluxes to adducts of E2 and E1 with DNA adducts in the network model considering transcripts encoding enzymes in biotransformation of E2/E1 identified by multiple linear regression models using stepwise forward selection as detailed in Online Resource 7. For each model, the number (*n*) of observations (O) contributing to the final model, the adjusted coefficient of determination (*R*^2^), and the observations (i.e., biospecimens) to exVAR ratio (O/exVAR) after forward selection of variables is given

To identify key reactions influencing metabolic fluxes to adducts of estrogens with DNA, multiple linear regression models using fluxes to adducts of estrogens with DNA as dependent variables and levels of E2, E1, and levels of transcripts encoding enzymes involved in estrogen biotransformation serving as flux constraints as exVARs were computed. Metabolic fluxes to adducts of E2 and E1 with DNA were positively influenced by levels of E1 and levels of *CYP1B1* as well as negatively influenced by levels of *GSTP1* and *NQO1* (all *P* < 0.05, Fig. 5).

Fluxes to adducts of E2 with DNA were further influenced positively by levels of E2 and *SULT1E1* and negatively by *SULT1A2* (all *P* < 0.05). E1-DNA were further influenced negatively (*P* < 0.05) by *SULT1A1* and *COMT* (Fig. 5).

#### Influence of breast cancer risk factors on metabolic fluxes to adducts of estrogens with DNA

**Fig. 6:**
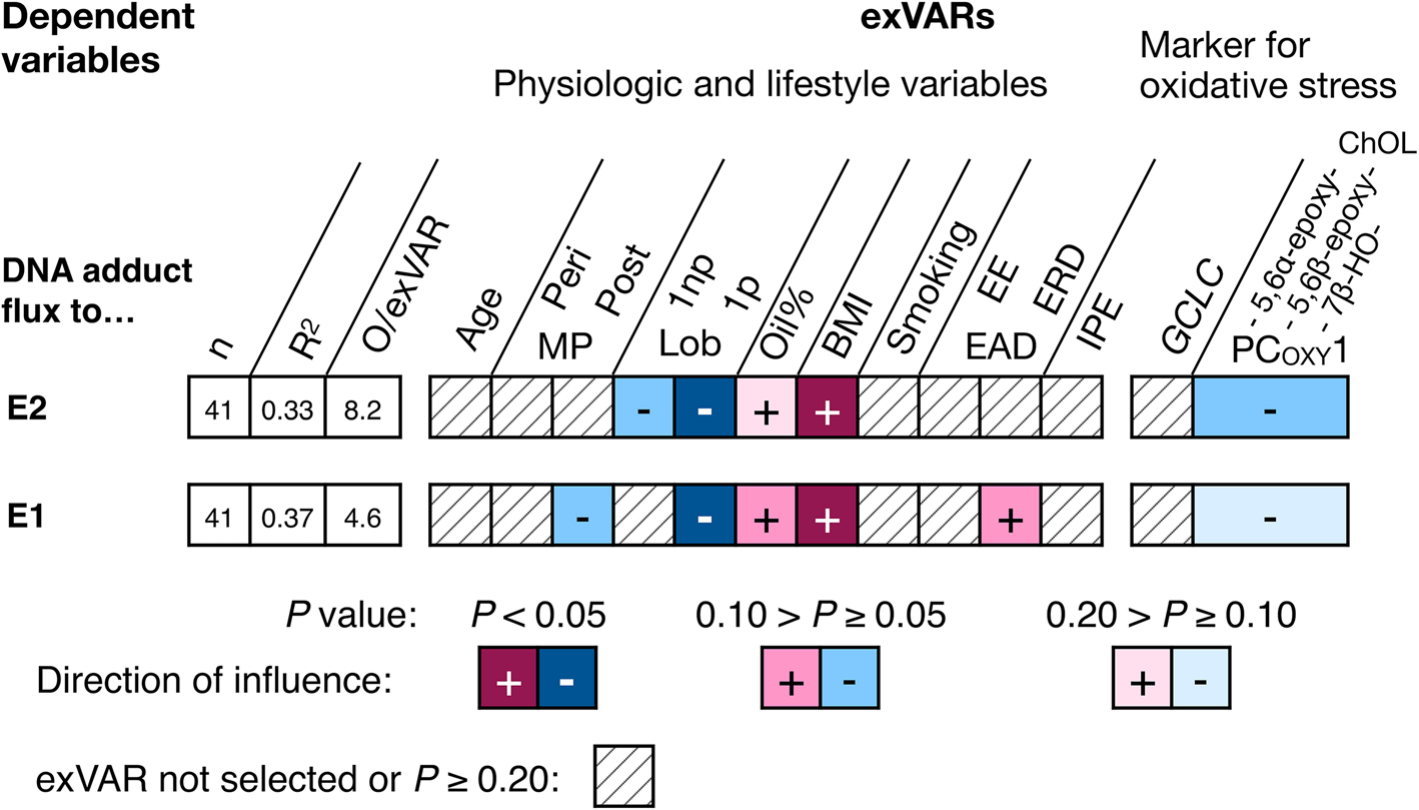
Influence of various exVARs on levels of calculated fluxes to adducts of E2 and E1 with DNA adducts in the network model considering breast cancer risk factors and markers for (oxidative) cellular stress identified by multiple linear regression models using stepwise forward selection as detailed in Online Resource 7. For each model, the number (*n*) of observations (O) contributing to the final model, the adjusted coefficient of determination (*R*^2^), and the observations (i.e., biospecimens) to exVAR ratio (O/exVAR) after forward selection of variables is given. *EAD* estrogen-active drug, *EE* ethinyl-E2, *ERD* E2-releasing drugs, *IPE* intake of dietary supplements containing phytoestrogens, *Lob 1np* lobule type 1 of nulliparous women, *Lob 1p* lobule type 1 of parous women, *periMP* perimenopausal, *postMP* postmenopausal

Levels of E2 and E1 as well as of transcripts encoding enzymes involved in estrogen biotransformation were used as flux constraints. Thus, these variables are mathematically related to the modeled fluxes. Therefore, these variables may not be tested with other variables as exVARs in MRLMs identifying exVARs influencing metabolic fluxes to adducts of estrogens with DNA. The remaining exVARs included breast cancer risk factors, intake of dietary supplements containing phytoestrogens, one short-term (levels of transcripts of *GCLC*) and one long-term marker for oxidative stress each, i.e., principal component of levels of oxyChOLs (PC_OXY_1) known to be formed exclusively by oxidative stress (Iuliano 2011), and general data regarding age, menopausal status, parity, and breast physiology (Fig. 6).

In addition to its indirect influence via influence on key flux constraints (i.e., levels of estrogens, levels of *CYP1B1* and *SULT1A1*), BMI influenced positively (*P* < 0.05) metabolic fluxes to adducts of E2 and E1with DNA (Fig. 6). Furthermore, fluxes to adducts of E2 and E1 with DNA were influenced negatively (*P* < 0.05) by the lobule-type characteristic for parous women after age-related regression of the breast glandular tissue. In addition, fluxes to adducts of E2 with DNA were influenced borderline positively (0.10 > *P* ≥ 0.05) by marker of long-term oxidative stress (PC_OXY_1, positive, since negative influence of negative values) and the intake of E2-releasing drugs, respectively.

One limitation of this study is that lifestyle factors, for which an impact on breast cancer risk and on circulating levels of E2 has been reported (alcohol consumption, physical activity, body fatness, (World Cancer Research Fund 2018; Hirko et al. 2014; Ennour-Idrissi et al. 2015; Tin Tin et al. 2020) could not be considered, because they were not part of the questionnaire (physical activity, body fatness) or were not provided consistently by the participants (alcohol consumption). Furthermore, the World Cancer Research Fund (2018) observed dietary habits which may alter breast cancer risk. Complementary studies on the impact of the representative food constituents (fiber, saturated as well as unsaturated fatty acids, calcium, and others) on levels of estrogens and/or inter- and intracellular signaling in glandular breast tissues are desirable but hitherto not available.

### Relevance

Whereas metabolic fluxes to estrogen metabolites within the network model were validated, metabolic fluxes to DNA adducts could not be validated because of the lack of data. Furthermore, variables affecting the lifetime of DNA adducts (such as enzymes involved in repair of DNA adducts) were not considered in the metabolic network. Thus, the modeled metabolic fluxes to adducts of estrogens with DNA represent the putative formation and not necessarily the prevalence of these adducts.

The metabolic network was based on transcript levels which do not in all cases represent the activities of enzymes or the amounts of active proteins. With experimental data an optimized solution can be found by the modeling of the whole network with YANAsquare calculating the actual flux strengths of the involved pathways in such a way that the error to the estimated enzyme activities is minimized. Metabolite measurements for nucleotide metabolites and carbohydrates indicate that the residual error in flux strength estimates is only 10% (Cecil et al. 2015). In general, marker transcripts indicating ESR1 activation, proliferation and cellular/oxidative stress were chosen due to their known regulation at mRNA level, yet other effects on protein and/or activity levels cannot be excluded.

**Fig. 7:**
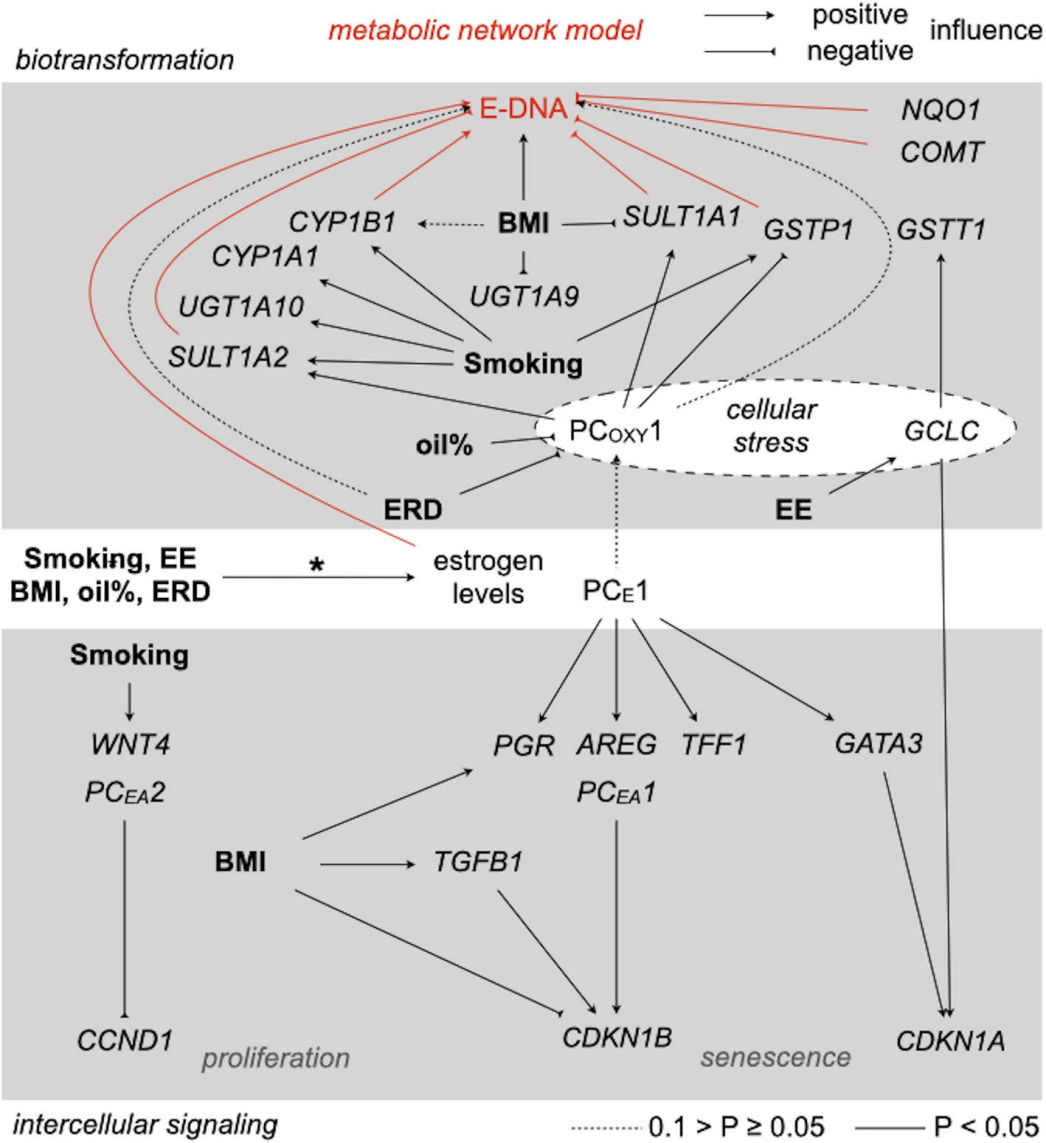
Influence of breast cancer risk factors (bold font) on cell proliferation and (de)activation of (pre)genotoxic estrogens identified by multiple linear regression. *Influence identified in Pemp et al. (2020). Estrogen levels as well as levels of *AREG*, *PGR*, *TFF1*, and of *WNT4* were tested as their principal components PC_E_1 as well as PC_EA_1 and PC_EA_2, respectively (Online Resource 7). Red: flux constraints significantly influencing metabolic fluxes to adducts of estrogens with DNA (E-DNA). For details which exVARs influence metabolic fluxes to adducts of E2 or E1 with DNA, see Figs. 5 and 6. *EE* ethinyl-E2, *ERD* E2-releasing drugs

Intratissue estrogen levels significantly influenced estrogen receptor activation, yet the overall influence on cell proliferation remains elusive (Fig. 7). Within the metabolic network model, estrogen levels did influence fluxes to adducts of estrogens with DNA, yet the sum of additional indirect effects via oxidative stress affecting deactivation of estrogen metabolites did not influence metabolic fluxes to adducts of estrogens with DNA significantly (Fig. 7). BMI, smoking, and intake of ethinyl-E2 or E2-releasing drugs all influenced estrogen levels (Pemp et al. 2020) and estrogen levels influenced fluxes to adducts of estrogens with DNA (Fig. 7). However, BMI was the only breast cancer risk factor influencing fluxes to adducts of estrogens with DNA, probably by increasing levels of *CYP1B1* and/or decreasing those of *SULT1A2* in addition to increasing estrogen levels (Fig. 7). The total influence of the BMI on proliferation cannot be resolved without further bioinformatic modeling.

Smoking did not influence fluxes to adducts of estrogens with DNA, possibly due to inducing transcript levels of both key enzymes involved in activation (*CYP1B1*) and deactivation (*GSTP1*, *SULT1A2*) of reactive estrogen metabolites (Fig. 7). Likewise, possible indirect influence of intake of estrogen-active drugs on estrogen biotransformation via oxidative stress did not affect modeled fluxes to adducts of estrogens with DNA (Fig. 7). Of note, enzymes involved in estrogen biotransformation influenced by smoking and/or estrogen-active drugs (CYP1A1, CYP1B1, UGT1A10, SULT1A2, GSTP1, SULT1A2) are involved in the (de)activation of other carcinogens as well. Therefore, lack of effect on fluxes to adducts of estrogens with DNA does not exclude an influence of these breast cancer risk factors on the (de-)activation of other carcinogens within the human breast.

## Supplementary Information

Below is the link to the electronic supplementary material.

Supplementary file1 (PDF 3213 KB)

Supplementary file2 (PDF 165 KB)

Supplementary file3 (PDF 659 KB)

Supplementary file4 (PDF 117 KB)

Supplementary file5 (PDF 785 KB)

Supplementary file6 (PDF 163 KB)

Supplementary file7 (PDF 1315 KB)

Supplementary file8 (PDF 112 KB)

## Data Availability

The datasets generated during and/or analyzed during this study are included in this published article [and its supplementary information files] or are available from the corresponding author on reasonable request.
