# Supplementary material for: Influence of breast cancer risk factors on proliferation and DNA damage in human breast glandular tissues: role of intracellular estrogen levels, oxidative stress and estrogen biotransformation
Source: Arch Toxicol. 2021 Dec 18;96(2):673–87. doi: 10.1007/s00204-021-03198-7 (PMC8837527; doi:10.1007/s00204-021-03198-7)
Supplement: Supplementary file 1 — Supplementary file1 (PDF 3213 KB) [file 204_2021_3198_MOESM1_ESM.pdf]

Archives of Toxicology

Influence of breast cancer risk factors on proliferation and DNA damage in human breast glandular tissues: role of intracellular estrogen levels, oxidative stress and estrogen biotransformation

Juliane Wunder, Daniela Pemp, Alexander Cecil, Maryam Mahdiani, René Hauptstein, Katja Schmalbach, Leo N. Geppert, Katja Ickstadt, Harald L. Esch, Thomas Dandekar, Leane Lehmann*

***Corresponding author:** Prof. Dr. Leane Lehmann, Chair of Food Chemistry, University of Würzburg, Am Hubland, D-97074 Würzburg, Germany. Phone: +49 931 318-5481.
Email: leane.lehmann@uni-wuerzburg.de.

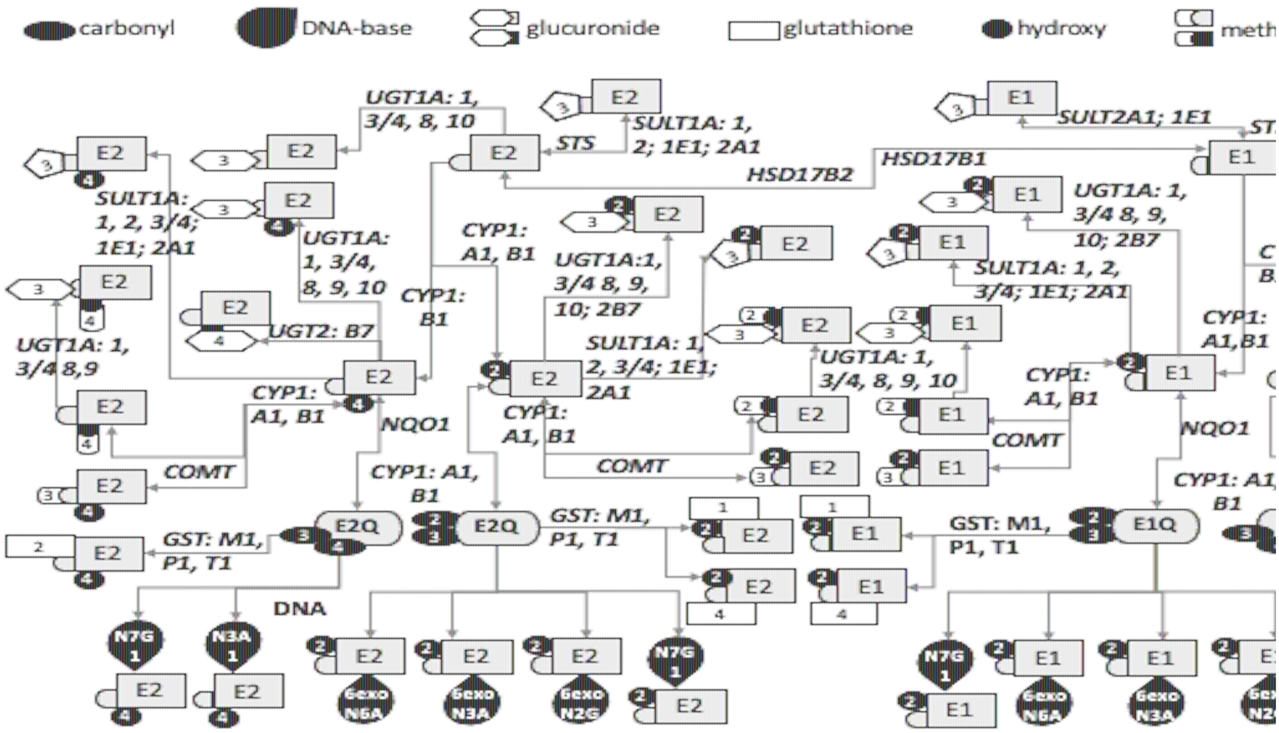

Online Resource 1 Biotransformation of E2 and E1 in human breast glandular tissues resulting in the formation of quinoid metabolites and subsequently in formation of adducts with DNA bases guanine (G) and adenine (A).

E2 and E1 are hydroxylated by CYPs to catechols and subsequently to electrophilic quinones (Qs) which react with nucleophilic centers of DNA bases of G (N3, N7) and A (N3, N6) to form DNA adducts. Quinone formation can be circumvented by (i) reduction of quinones by NADPH quinone dehydrogenase 1, (NQO1), (ii) methylation of catechols by catechol-*O*-methyl transferase (COMT) or (iii) conjugation of E2 and E1; i.e. sulfonation and glucuronidation by sulfotransferases (SULTs) and UDP-glucuronosyltransferases (UGTs), respectively. In general, CYPs, SULTs and UGTs accept various substrates for hydroxylation, sulfonation or glucuronidation, respectively, resulting in overlapping substrate specificities. Interconversion between E2 and E1 and their metabolites is catalyzed by hydroxysteroid 17-beta dehydrogenases (HSD17Bs), yet in contrast to the difference in hormonal activity between E2 and E1 there is no indication that E1Qs are less genotoxic than E2Qs.

Depicted (iso)enzymes, substrates and products represent the current understanding of E2 biotransformation in the mammary gland of women without breast cancer and are based on Online Resource 3 and published data summarized in Pemp et al. 2019).

Reference

Pemp D, Kleider C, Schmalbach K, et al. (2019) Qualitative and quantitative differences in estrogen biotransformation in human breast glandular and adipose tissues: implications for studies using mammary biospecimens. Arch Toxicol 93:2823-2833. https://doi.org/10.1007/s00204-019-02564-w
