# Supplementary material for: Influence of breast cancer risk factors on proliferation and DNA damage in human breast glandular tissues: role of intracellular estrogen levels, oxidative stress and estrogen biotransformation
Source: Arch Toxicol. 2021 Dec 18;96(2):673–87. doi: 10.1007/s00204-021-03198-7 (PMC8837527; doi:10.1007/s00204-021-03198-7)
Supplement: Supplementary file 2 — Supplementary file2 (PDF 165 KB) [file 204_2021_3198_MOESM2_ESM.pdf]

Archives of Toxicology

Influence of breast cancer risk factors on proliferation and DNA damage in human breast glandular tissues: role of intracellular estrogen levels, oxidative stress and estrogen biotransformation

Juliane Wunder, Daniela Pemp, Alexander Cecil, Maryam Mahdiani, René Hauptstein, Katja Schmalbach, Leo N. Geppert, Katja Ickstadt, Harald L. Esch, Thomas Dandekar, Leane Lehmann*

***Corresponding author:** Prof. Dr. Leane Lehmann, Chair of Food Chemistry, University of Würzburg, Am Hubland, D-97074 Würzburg, Germany. Phone: +49 931 318-5481. Email: leane.lehmann@uni-wuerzburg.de.

Online Resource 2 Composition of principal components (PCs) calculated in R using levels of 17β-estradiol (E2), estrone (E1), and E1-sulfate (-S) in human breast glandular tissue derived from 41 women without breast cancer and resulting values of principal component (PC_E1) used in multiple linear regression models. Percentages of variations explained by the PCs are provided and eigenvectors of prominent variables influencing the PCs are indicated with bold characters.
Breast glandular tissue levels of E2, E1, E1-S, 2-methoxy (2-MeO-) E1 and E1-glucuronide (-G) of women without breast cancer used for calculation and validation of metabolic network (E2, E1, 2-MeO-E1, E1-G) and multiple linear regression models (E2, E1, E1-S) have been published previously (Pemp et al. 2020). Estrogen tissue levels of women No. 4, 21, 27, 34, 37 and 47 (Pemp et al. 2020) have not been considered in this study because of exclusion from the network model or missing values in explanatory variables.
*used in multiple linear regression models on levels of transcripts of markers for ESR activation and proliferation, levels of transcripts encoding enzymes in biotransformation of E2 and E1 as well as markers for (oxidative) cellular stress. Criteria for choosing PCs are presented in Online Resource 7.

Variable	Eigenvectors		
	PC_E1*	PC_E2	PC_E3
E1	**0.60**	-0.4	**-0.7**
E1S	**0.50**	-0.4	**0.7**
E2	**0.60**	**0.9**	<0.1
% of variation explained	73	21	6

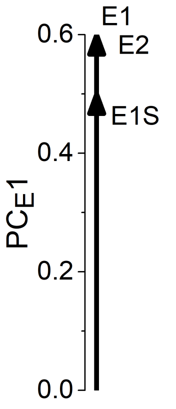

**No.**	**PC_E1**	**No.**	**PC_E1**
1	-0.6	23	-1.4
2	0.7	24	1.2
3	-0.3	25	3.0
5	-1.2	27	-0.8
6	-0.9	28	0.5
7	-1.0	29	-1.0
8	-0.7	30	1.0
9	-1.0	31	2.5
10	2.5	32	-0.6
11	3.6	33	-0.9
12	1.7	34	-1.0
13	-1.5	35	-1.2
14	-1.4	36	-1.2
15	2.9	37	-1.2
16	-1.1	38	0.7
17	2.3	39	1.4
18	-1.4	40	-1.4
19	-1.1	41	-0.9
20	-0.8	42	0.5
21	1.3	43	-1.3
22	<0.1		

Reference

Pemp D, Geppert LN, Wigmann, C, Kleider C, Hauptstein R, Schmalbach K, Ickstadt K, Esch HL, Lehmann L (2020). Influence of breast cancer risk factors and intramammary biotransformation on estrogen homeostasis in the human breast. Arch Toxicol 94:3013–3025. https://doi.org/10.1007/s00204-020-02807-1
