# Supplementary material for: Influence of breast cancer risk factors on proliferation and DNA damage in human breast glandular tissues: role of intracellular estrogen levels, oxidative stress and estrogen biotransformation
Source: Arch Toxicol. 2021 Dec 18;96(2):673–87. doi: 10.1007/s00204-021-03198-7 (PMC8837527; doi:10.1007/s00204-021-03198-7)
Supplement: Supplementary file 3 — Supplementary file3 (PDF 659 KB) [file 204_2021_3198_MOESM3_ESM.pdf]

Influence of breast cancer risk factors on proliferation and DNA damage in human breast glandular tissues: role of intracellular estrogen levels, oxidative stress and estrogen biotransformation

Juliane Wunder, Daniela Pemp, Alexander Cecil, Maryam Mahdiani, René Hauptstein, Katja Schmalbach, Leo N. Geppert, Katja Ickstadt, Harald L. Esch, Thomas Dandekar, Leane Lehmann*

***Corresponding author:** Prof. Dr. Leane Lehmann, Chair of Food Chemistry, University of Würzburg, Am Hubland, D-97074 Würzburg, Germany. Phone: +49 931 318-5481. Email: leane.lehmann@uni-wuerzburg.de.

Online Resource 3 Transcript levels used for calculation of the network model and in multiple linear regression models and composition and values of the principal components (PCs) of transcript levels used in multiple linear regression models.

Part A Transcript levels of markers for proliferation and signaling, of enzymes involved in biotransformation and of markers of oxidative stress. Levels of transcripts involved in biotransformation and included in the metabolic network model are shown for the 44 biospecimens of which data have been used in the calculations of the metabolic network model and the multiple linear regression models; levels of transcripts used exclusively in multiple linear regression models are shown for the 41 biospecimens which provided the respective data. Transcript levels were determined by TaqMan® low density array (TLDA) and commercially available TaqMan®-assays.

* not used in forward selected multiple regression models, because of missing values in explanatory variables

n.u.: not used/value not calculated.

<, below LOD or below LOQ: Calculated with values of LOD or LOQ of the respective transcripts in the network calculation and in use as dependent variable. Below the used replacements:

CCND1 LOD 5.34 x 10^{-8}; *CYP1A1* LOD 5.34 x 10^{-8}; *GSTM1* LOD 5.67 x 10^{-8} , *SULT1E1* LOQ 6.83 x 10^{-6}; *SULT2A1* LOD 5.34 x 10^{-8}, LOQ 6.83 x 10^{-6}; *UGT1A1* LOD 5.67 x 10^{-8}, LOQ 1.81 x 10^{-6}; *UGT1A3/4* LOD 5.34 x 10^{-8}, LOQ 6.83 x 10^{-6}; *UGT1A8* LOD 5.34 x 10^{-8}, LOQ 6.83 x 10^{-6}, *UGT1A9* LOD 5.67 x 10^{-8}, LOQ 1.81 x 10^{-6}; *UGT1A10* LOD 5.67 x 10^{-8}; *UGT2B7* LOD 5.34 x 10^{-6}, LOQ 6.83 x 10^{-6}.

	proliferation and signaling											biotransformation and oxidative stress					
					ESR								CYP		HSD17B		
No.	*AREG*	*CCND1*	*CDKN1A*	*CDKN1B*	*1*	*2*	*GATA3*	*PGR*	*TFF1*	*TGFB1*	*WNT4*	*COMT*	*1A1*	*1B1*	*1*	*2*	*GCLC*
1	0.62894	4.13532	0.66434	3.45336	2.07053	0.02031	5.20898	0.40000	0.14814	1.54756	0.09910	0.58035	0.00096	0.40304	0.00622	0.29792	0.77378
2	5.37774	<LOD	0.45250	2.96493	1.84165	0.00915	3.06099	0.26400	0.54261	0.80051	0.41581	0.47100	0.04380	0.96393	0.00151	0.36148	0.78187
3	0.93952	1.36888	1.27015	4.23981	0.92530	0.01630	3.81055	0.26499	0.04941	0.96594	0.13956	0.37700	0.02010	0.57236	0.00348	0.23037	1.09000
4*	n.u.	n.u.	n.u.	n.u.	n.u.	n.u.	n.u.	n.u.	n.u.	n.u.	n.u.	0.22300	0.00124	3.09728	0.00110	0.21628	n.u.
5	0.73255	1.88426	3.94220	4.74695	2.16895	0.04225	6.11775	0.16600	0.01908	1.11651	0.06880	0.55800	0.00011	1.05263	0.02272	0.33217	1.05922
6	0.34700	<LOD	1.80625	5.24157	2.55974	0.01130	12.2185	0.21479	0.26702	1.95884	0.04395	0.55400	0.00419	0.78241	0.00390	0.25279	0.73000
7	0.29382	2.88786	0.99516	7.27527	1.48968	0.02604	2.52275	0.13000	0.04022	0.92274	0.09590	0.41000	0.00225	0.60794	0.01507	0.32086	1.27810
8	0.31710	<LOD	1.40444	5.67256	3.07375	0.11034	7.19503	0.44627	0.00578	3.02723	0.07700	0.69112	0.00060	0.92787	0.01492	0.12175	1.50212
9	0.59460	<LOD	1.54864	2.70570	4.57572	0.02958	2.80889	0.19100	0.02080	0.88087	0.03853	3.73000	0.00296	2.02090	0.00656	0.05194	1.87125
10	2.46530	2.36638	1.11613	4.33959	2.54080	0.02050	4.32968	0.88500	0.63101	1.71316	0.08570	0.82100	0.00318	1.54384	0.00132	0.76663	1.13955
11	1.86348	<LOD	0.33425	1.86090	0.54829	0.00580	2.32302	0.23651	0.59874	0.43347	0.24249	0.77100	0.00003	0.83625	0.00099	0.14783	0.43167
12	2.25793	2.32302	0.36173	2.09072	0.69352	0.00630	3.89872	0.85976	1.81252	0.64932	0.13612	0.48300	0.00124	0.67971	0.00063	0.27624	0.85323
13	1.21419	1.25701	1.22774	3.72696	4.06992	0.00816	5.40015	0.23100	0.21991	1.81252	0.04962	1.78633	0.00096	0.95396	0.00046	0.30355	0.81282
14	0.19957	2.20381	0.47665	1.34630	1.51362	0.00478	2.69260	0.04041	0.02362	0.82473	0.02820	0.19683	0.00035	3.13399	0.00193	0.20791	0.34600
15	2.36362	5.66078	1.09657	7.32079	0.79664	0.03233	3.33804	0.44200	1.29146	1.82513	0.13774	1.09000	0.04290	0.87418	0.02018	0.14711	1.70409
16	3.40581	1.04608	0.23882	3.57266	1.26488	0.00802	2.58829	0.20041	0.18816	0.76684	0.24098	0.62503	0.00089	1.41716	0.00049	0.15156	0.57794
17	0.75315	1.97657	0.36023	3.45575	0.79499	0.01219	2.73208	0.24879	0.08944	0.90313	0.05220	0.43200	0.00063	0.99585	0.00087	0.30020	0.51192
18	0.18175	<LOD	0.60249	2.04344	2.10672	0.00943	5.44526	0.15325	0.04175	0.47303	0.04910	1.13000	0.00012	0.37919	0.00269	0.05880	0.63000
19	2.15696	6.15603	0.55594	6.44866	3.23328	0.00930	5.30738	0.20533	0.02462	2.00277	0.07716	5.95046	0.01950	2.37347	0.00433	0.36198	1.06000
20	1.72309	2.37019	0.22798	3.92040	1.62338	0.00987	2.94241	0.23734	0.11574	1.32134	0.09414	0.57197	0.11400	0.69544	0.00351	0.41783	0.70907
21	4.57890	1.66209	0.39420	4.13819	2.32141	0.00686	3.26481	0.98800	11.6721	1.21672	0.09233	0.79443	0.00051	2.18102	0.00041	0.25331	0.78295
22	0.38582	1.12039	1.00905	4.78660	1.68529	0.01917	4.02224	0.41600	0.27913	1.61888	0.07450	0.79200	0.00042	2.15248	0.00129	0.11447	1.01255
23	0.58927	<LOD	0.39202	3.28752	4.78328	0.00955	3.59502	0.13688	0.01147	0.84382	0.04873	1.03097	0.00183	0.45597	0.00068	0.38236	0.80441
24	2.20840	0.80608	0.72497	4.29602	0.91447	0.00812	5.23794	0.30300	0.50000	1.13524	0.15198	0.28900	0.00003	0.72598	0.00053	0.06556	0.52300
25	1.27987	1.22010	0.23085	1.91986	0.53071	0.00416	1.85446	0.20405	0.54450	0.40641	0.30313	0.52600	0.00133	1.30044	0.00020	0.41123	0.48000
26*	n.u.	n.u.	n.u.	n.u.	n.u.	n.u.	n.u.	n.u.	n.u.	n.u.	n.u.	0.77700	0.00136	1.38407	0.00071	0.58524	n.u.
27	0.36704	<LOD	0.92787	4.04461	2.94037	0.01727	5.68831	0.13603	0.00605	1.34257	0.04617	1.06000	0.00075	1.59549	0.00317	1.46713	1.07848
28	1.33700	2.04627	0.51584	6.14750	1.11806	0.00875	3.80264	0.61000	0.07647	1.22519	0.27932	0.78200	0.00007	0.44751	0.00075	0.24759	0.68500
29	0.90125	2.40161	0.30418	3.14269	1.13210	0.00717	3.57514	0.23300	0.13650	0.69882	0.11203	0.86600	0.02435	2.30537	0.00034	0.20278	0.94344
30	1.63241	2.23457	1.32042	5.09471	3.69097	0.02334	12.8349	0.17200	0.26098	0.81677	0.22846	0.89900	0.00073	0.20863	0.00682	0.35282	1.12506
31	2.39994	3.04407	2.52450	5.45659	1.20748	0.03701	3.10372	0.27800	0.09176	1.62676	0.09106	0.71698	0.00410	1.09885	0.01061	0.64618	1.60993
32	0.50909	1.67597	1.14155	2.75872	0.99861	0.01464	5.71202	0.27567	0.11720	0.91955	0.18416	0.45125	0.00165	1.12350	0.00389	0.15921	1.03886
33	1.19003	5.64902	0.86995	4.11531	1.99723	0.01801	3.17334	0.15294	0.02253	1.28165	0.08693	0.61000	0.00247	0.80329	0.00716	0.49243	0.81451
34	0.34796	0.93952	0.55287	4.12102	1.70764	0.00971	5.25613	0.11250	0.08455	1.19251	0.04704	0.60249	0.01286	0.32331	0.00058	0.31997	0.71797
35	2.49666	<LOD	1.28967	4.81655	1.34537	0.04910	3.36592	0.20964	2.05623	1.43594	0.16800	0.71600	0.34200	1.00626	0.01414	0.45188	0.93400
36	0.78513	<LOD	0.23882	2.40994	0.84792	0.00783	2.36199	0.09520	0.02631	0.74226	0.06452	0.46200	0.00002	0.60584	0.00010	0.75471	0.79996
37	0.57955	1.38607	1.20748	5.21259	2.53678	0.02495	2.29740	0.20800	0.06138	1.99032	0.07500	0.56900	<LOD	1.17772	0.02057	0.10366	1.17691
38	1.42109	0.58277	0.66388	2.82255	0.83047	0.00833	2.48802	0.62900	0.16054	2.24700	0.12869	1.26400	0.00011	1.65061	0.00386	0.21991	0.94083
39	1.65749	8.12856	0.53145	6.14750	2.38337	0.00496	4.52526	1.43000	2.71886	0.75733	0.12234	1.18000	0.00054	1.78757	0.00123	0.91257	0.81790
40	0.93562	1.91853	2.38667	4.89056	0.58520	0.01320	3.33804	0.21600	0.34009	1.15669	0.66526	0.80200	0.00081	1.36415	0.00458	0.48397	1.56374
41	0.58398	4.07839	1.68646	3.80527	15.4121	0.00527	16.0666	0.28401	0.10518	0.87721	0.05278	0.96326	0.18492	2.24233	0.00351	0.06731	0.97942
42	3.82909	5.97525	1.76663	9.98664	1.16151	0.00857	4.22221	1.01255	8.53860	3.13182	0.15432	1.63580	0.00040	1.44894	0.00155	0.19833	0.86514
43	1.59169	2.05683	1.00679	5.25939	3.88609	0.00816	20.0375	0.41170	1.70792	1.38940	0.11413	1.16949	0.00082	1.49542	0.00042	0.40475	1.52309
44*	n.u.	n.u.	n.u.	n.u.	n.u.	n.u.	n.u.	n.u.	n.u.	n.u.	n.u.	0.87055	0.00108	8.32817	0.00331	0.13149	n.u.

	biotransformation and oxidative stress															
	GST					SULT					UGT					
No.	*M1*	*P1*	*T1*	*NQO1*	*STS*	*1A1*	*1A2*	*1A3/4*	*1E1*	*2A1*	*1A1*	*1A3/4*	*1A8*	*1A9*	*1A10*	*2B7*
1	<LOD	16.5000	0.56999	2.23268	0.49931	0.00549	0.01830	0.06134	0.00305	<LOD	<LOD	<LOD	0.00008	0.00011	0.00021	<LOQ
2	<LOD	31.4000	0.53071	3.93826	0.42632	0.00529	0.01160	0.08627	0.00172	<LOQ	0.00008	<LOD	<LOQ	0.00023	0.00024	<LOQ
3	<LOD	35.3000	0.82645	2.32000	0.36450	0.00401	0.00910	0.07433	0.00163	<LOD	0.00006	<LOQ	0.00005	0.00010	0.00015	<LOQ
4*	<LOD	23.2000	0.37893	1.81541	0.38770	0.00393	0.01386	0.11391	<LOQ	<LOD	0.00059	<LOD	0.00003	0.00104	0.00168	<LOQ
5	0.21353	28.2000	0.64305	2.71401	0.59584	0.03612	0.05909	0.19238	0.00010	<LOQ	0.00015	<LOD	<LOQ	0.00120	0.00071	<LOD
6	<LOD	27.1000	1.56482	4.88000	0.62894	0.00402	0.01193	0.09889	0.00190	<LOQ	0.00008	0.00010	0.00013	0.00010	0.00034	<LOQ
7	<LOD	27.9000	0.84792	2.85000	0.67689	0.00707	0.01550	0.06342	0.00004	<LOD	<LOQ	<LOQ	<LOQ	0.00019	0.00037	<LOQ
8	0.19946	11.5000	1.77399	1.34000	0.56880	0.01015	0.03050	0.21154	0.00103	<LOD	<LOD	<LOD	0.00005	0.00034	0.00012	<LOQ
9	<LOD	20.4000	0.73918	3.45402	2.32785	0.00285	0.02736	0.38400	0.00067	<LOD	0.00066	0.00029	0.00005	0.00014	0.00021	<LOD
10	<LOD	29.9000	0.63574	3.50000	1.31000	0.01149	0.01640	0.65726	0.00196	<LOQ	<LOD	<LOD	0.00006	0.00012	0.00040	<LOQ
11	<LOD	14.2000	0.26207	1.14000	0.26080	0.00359	0.00692	0.04860	0.00169	<LOD	<LOD	<LOD	<LOQ	0.00004	0.00048	<LOQ
12	<LOD	18.3000	0.46911	1.58000	0.58479	0.00362	0.01025	0.06887	0.00014	<LOD	0.00009	<LOD	<LOD	0.00016	0.00009	<LOQ
13	<LOD	25.6000	0.50174	1.97000	0.44350	0.00406	0.02781	0.11574	0.00047	<LOD	0.00004	<LOD	<LOD	0.00028	0.00033	0.00005
14	<LOD	36.3000	0.16009	1.98083	0.11500	0.00451	0.17776	0.14968	0.00121	<LOD	<LOD	<LOD	0.00003	0.00020	0.00082	<LOQ
15	<LOD	38.0000	2.10818	6.09276	0.78731	0.01360	0.03879	0.13416	0.00004	<LOD	0.00023	<LOD	0.00002	0.00008	0.00012	0.00016
16	0.12253	21.6000	0.00001	1.94000	2.19314	0.00466	0.00793	0.09280	0.00039	<LOD	<LOD	<LOD	<LOD	0.00013	<LOD	<LOQ
17	<LOD	21.7000	0.39039	0.58640	1.08749	0.00335	0.01120	0.24065	0.00020	<LOD	0.00044	<LOD	<LOQ	0.00022	0.00017	<LOD
18	<LOD	30.9000	0.41581	3.68000	0.27319	0.00572	0.00725	0.18276	0.00179	<LOD	0.00028	<LOD	0.00004	0.00029	0.00014	<LOQ
19	<LOD	32.5000	0.00071	1.39785	11.70457	0.00280	0.00594	0.52778	0.00002	<LOD	0.00030	<LOD	<LOQ	0.00034	0.00036	<LOD
20	<LOD	33.4000	0.41151	1.92242	0.75367	0.00220	0.01830	0.04058	0.00004	<LOD	0.00012	<LOD	<LOQ	0.00051	0.00065	0.00027
21	0.05769	24.4000	0.78187	2.13059	1.17283	0.00319	0.02012	0.07290	0.00068	<LOD	<LOD	<LOD	0.00004	0.00012	0.00011	<LOQ
22	0.15526	30.4000	0.71598	1.22536	0.62028	0.00341	0.00872	0.10912	0.00500	<LOD	0.00038	<LOQ	0.00001	0.00023	0.00048	<LOQ
23	0.00036	28.2000	0.45031	2.79000	0.38800	0.00319	0.01601	0.16188	0.00272	<LOD	0.00030	<LOD	0.00006	0.00032	0.00028	<LOQ
24	<LOD	23.3000	0.90941	1.23000	0.35824	0.00222	0.00430	0.32376	0.00169	<LOD	0.00005	<LOD	0.00001	0.00010	0.00010	0.00008
25	<LOD	23.1000	0.57955	0.71100	0.33798	0.00276	0.00472	0.06916	0.00003	<LOD	<LOD	<LOD	<LOQ	0.00013	0.00036	<LOQ
26*	<LOD	18.2000	0.48736	1.31670	0.67050	0.00379	0.00771	0.08219	0.00004	<LOQ	0.00009	<LOD	<LOQ	0.00012	0.00010	<LOQ
27	<LOD	35.2000	1.62902	4.46000	0.68113	0.00397	0.02120	0.09652	0.00090	<LOQ	0.00055	<LOQ	0.00008	0.00048	0.00076	<LOQ
28	<LOD	17.6000	0.41581	1.33000	0.28600	0.00460	0.00570	0.23277	0.00095	<LOD	0.00006	<LOD	0.00002	0.00020	0.00009	<LOD
29	<LOD	17.0000	0.73918	1.00204	0.49896	0.00237	0.01081	0.08340	0.00005	<LOD	<LOD	<LOD	<LOD	0.00003	0.00018	<LOD
30	<LOD	29.3000	0.00160	2.64000	0.36900	0.00247	0.03930	0.25402	0.00237	<LOD	<LOD	<LOD	<LOD	0.00019	<LOD	<LOQ
31	<LOD	18.7000	0.88209	2.56327	1.35000	0.00565	0.01253	0.12500	0.00012	<LOD	<LOD	<LOD	0.00005	0.00007	0.00004	<LOQ
32	<LOD	29.7955	0.69255	3.52139	0.47041	0.00418	0.01115	0.09903	0.00048	<LOD	<LOD	<LOD	0.00005	<LOD	<LOD	0.00020
33	<LOD	23.4000	0.00034	1.72000	0.91828	0.00255	0.00889	0.07270	0.00005	<LOD	<LOD	<LOQ	<LOQ	<LOD	0.00015	<LOD
34	<LOD	21.5412	0.51406	4.11107	0.59378	0.00160	0.00839	0.04704	0.00178	<LOD	0.00037	<LOQ	<LOQ	0.00039	0.00066	<LOQ
35	<LOD	26.3000	0.81113	2.27000	0.55633	0.00851	0.01360	0.23619	0.00024	<LOD	<LOD	<LOD	<LOD	0.00015	0.00037	<LOD
36	<LOD	22.6000	0.51228	0.24300	0.16700	0.00201	0.00252	0.06769	0.00003	<LOD	0.00019	<LOD	0.00002	0.00011	0.00019	<LOD
37	<LOD	23.3000	0.75158	3.61000	0.52400	0.00354	0.01750	0.13351	0.00057	<LOD	0.00007	0.00006	<LOQ	<LOD	<LOD	<LOQ
38	<LOD	31.0000	0.68113	2.66000	0.22700	0.00169	0.01246	0.09300	0.00036	<LOQ	<LOD	0.00043	0.00003	<LOQ	0.00002	<LOD
39	<LOD	21.8000	0.70515	1.33000	0.56600	0.00584	0.01064	0.15700	0.00154	<LOD	<LOD	<LOD	<LOQ	0.00001	0.00006	0.00002
40	<LOD	30.2000	1.50108	1.84724	0.32900	0.00444	0.00629	0.10006	0.00024	<LOQ	<LOD	<LOD	<LOD	<LOD	<LOD	<LOD
41	<LOD	27.2827	2.11404	3.06162	0.41639	0.00387	0.00920	0.13660	0.02272	<LOQ	<LOD	<LOD	0.00003	0.00003	<LOD	<LOQ
42	0.29171	16.1106	1.08749	1.90495	0.63156	0.00513	0.02339	0.07081	0.00007	<LOD	NA	<LOQ	<LOQ	0.00003	0.00003	<LOD
43	<LOD	21.6922	0.93068	5.39057	0.61833	0.00632	0.01103	0.07167	0.00046	<LOD	NA	<LOD	0.00002	<LOQ	0.00007	<LOQ
44*	0.00325	39.2667	1.53581	2.58999	0.38236	0.00266	0.01800	0.08339	0.00057	<LOD	NA	<LOQ	<LOD	<LOQ	0.00009	0.00158

Part B Composition of PCs calculated in R using transcript levels of AREG, PGR, TFF1 and WNT4 in human breast glandular tissue derived from 41 women without breast cancer and resulting values of principal components ($PC_{EA}1$ and 2) used in multiple linear regression models.

Percentages of variations explained by the PCs are provided and eigenvectors of prominent variables influencing the PCs are indicated with bold characters.

*used in multiple linear regression models describing levels of transcripts indicating ESR activation and proliferation as explanatory variables. Criteria for choosing PCs are presented in Online Resource 7. n.u.: not used/value not calculated.

Variable	Eigenvectors			
	PC_{EA} 1*	PC_{EA} 2*	PC_{EA} 3	PC_{EA} 4
AREG	**0.57**	0.26	**0.56**	**-0.54**
PGR	**0.54**	-0.30	**-0.71**	-0.32
TFF1	**0.59**	-0.26	0.23	**0.73**
WNT4	0.19	**0.88**	-0.35	0.26
% of	54	27	12	7

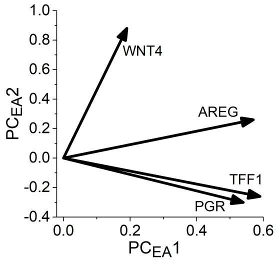

No.	PC_{EA} 1	PC_{EA} 2	No.	PC_{EA} 1	PC_{EA} 2
1	-0.5393	-0.4008	23	-1.1567	-0.4957
2	2.06054	3.07610	24	0.20455	0.39496
3	-0.6013	0.11719	25	-0.1586	1.40888
4*	n.u.	n.u.	26*	n.u.	n.u.
5	-1.0017	-0.3466	27	-1.2684	-0.5616
6	-1.0683	-0.6937	28	0.44921	0.88052
7	-1.2249	-0.2069	29	-0.6991	-0.0721
8	-0.6746	-0.6638	30	-0.2464	0.99834
9	-1.0691	-0.6269	31	0.04300	0.05824
10	1.31860	-0.6549	32	-0.6946	0.33480
11	0.09226	1.04731	33	-0.7802	-0.0994
12	1.56828	-0.4367	34	-1.2983	-0.5441
13	-0.6338	-0.4738	35	0.60652	0.49343
14	-1.5465	-0.6347	36	-1.1115	-0.2947
15	0.71830	0.08886	37	-0.9757	-0.3822
16	0.64109	1.45715	38	0.30343	-0.2462
17	-0.8486	-0.5585	39	2.54677	-1.3619
18	-1.3100	-0.6022	40	0.22988	4.02765
19	-0.2444	-0.0151	41	-0.8587	-0.6293
20	-0.3386	-0.0274	42	4.39476	-0.8914
21	5.43136	-1.5227	43	0.37165	-0.2712
22	-0.6294	-0.6677	44*	n.u.	n.u.
