# Supplementary material for: Influence of breast cancer risk factors on proliferation and DNA damage in human breast glandular tissues: role of intracellular estrogen levels, oxidative stress and estrogen biotransformation
Source: Arch Toxicol. 2021 Dec 18;96(2):673–87. doi: 10.1007/s00204-021-03198-7 (PMC8837527; doi:10.1007/s00204-021-03198-7)
Supplement: Supplementary file 4 — Supplementary file4 (PDF 117 KB) [file 204_2021_3198_MOESM4_ESM.pdf]

Archives of Toxicology

Influence of breast cancer risk factors on proliferation and DNA damage in human breast glandular tissues: role of intracellular estrogen levels, oxidative stress and estrogen biotransformation

Juliane Wunder, Daniela Pemp, Alexander Cecil, Maryam Mahdiani, René Hauptstein, Katja Schmalbach, Leo N. Geppert, Katja Ickstadt, Harald L. Esch, Thomas Dandekar, Leane Lehmann*

***Corresponding author:** Prof. Dr. Leane Lehmann, Chair of Food Chemistry, University of Würzburg, Am Hubland, D-97074 Würzburg, Germany. Phone: +49 931 318-5481. Email: leane.lehmann@uni-wuerzburg.de.

Online Resource 4 Determination of tissue levels of oxidation products of cholesterol by GC-MS/MS.

Unless indicated otherwise, solvents and reagents were obtained from Merck/Sigma-Aldrich (Germany), Carl-Roth (Germany), Alfa Aesar (Germany), Fisher Scientific (Germany), Riedel-de-Haen (Germany) at MS-grade.

Synthesis of deuterated oxy-ChOLs

Deuterated oxidation products of cholesterol (oxy-ChOLs-d6) were used as internal standards for the quantitation of oxyChOLs. Since oxy-ChOLs-d6 are not commercially available, they were synthesized by thermal oxidation (4 h, 160°C in an oven) of cholesterol-26,26,26,27,27,27-d6 (ChOL-d6, Medical Isotopes, USA). After addition of toluene, oxy-ChOLs-d6 were separated from ChOL-d6 on a Discovery SPE DSC-Si column (100 mg) using 2-propanol/n-hexane (15 + 30 v/v) as eluent. After evaporation of the eluents, oxy-ChOLs-d6 were quantified using non-deuterated oxy-ChOLs (Steraloids, USA) by GC-MS/MS as described below.

Sample preparation

100 mg frozen powdered breast adipose tissue (Pemp et al. 2019) was suspended in 10 ml of a mixture containing chloroform:methanol (2:1 v/v), 80 mg butyl hydroxytoluene and 100 µl of a mixture containing oxy-ChOLs-d6 (14,0 ng 5,6α-epoxy-ChOL; 11,1 ng 5,6β-epoxy-ChOL; 7,1 ng 7β-HO-ChOL). After extraction at room temperature for 1 h, 4 ml of a mixture of methanol and 0.9% NaCl solution (1:1 v/v) were added, vortexed and centrifuged at 4000 rpm for 5 min

(Universal 16, Hettich, Germany). The aqueous phase was discarded and the washing process was repeated. The chloroform phase was evaporated to dryness.

The dried residue was saponificated at room temperature in ethanolic potassium hydroxide (2 M) over night. After adding dichloromethane (10 ml) the mixture was vortexed and centrifuged at 4000 rpm for 5 min. The aqueous phase was discarded, and the organic phase washed twice with water. After drying with sodium sulphate until no more turbidity was observed the organic phase was evaporated to dryness and the residue dissolved in 1 ml of toluene.

Purification of the extract was achieved by SPE on a silica column (conditioned with n-hexane and equilibrated with toluene). Loaded extract was washed with n-hexane (2 ml) and n-hexane containing 0.5% 2-propanol (8 ml). OxyChOLs were eluted with 30% 2-propanol in n-hexane (5 ml). Samples were evaporated to dryness prior to derivatization.

Derivatization and analysis by GC-MS/MS

For derivatization, BSTFA/n-heptane (1:1) was added to the dried residues, transferred with a glass pipette into a GC vial and kept at room temperature for three hours.

For GC-MS/MS analysis of oxy-ChOLs a Varian 300-MS interfaced with a Varian 450-GC system (Bruker Daltonics, Germany) was used. Analytes were separated on a Supelco SLB-5ms fused silica, 30 m x 0.25 mm capillary column with 5% phenyl-, 95% methylpolysiloxan (0.25 µm) as stationary phase. Injection volume was up to 2 µl splitless. Helium (5.3; Linde, Germany) was used as carrier gas was at 1 ml/min. The injector temperature was 250°C, and the column oven was programmed at 200°C for 2 min, then ramped at 4°C/min to 315°C and held for 5 min. Electron impact ionization occurred at 70 eV with an interface temperature of 280°C and source temperature of 250°C. For detection multiple-reaction-monitoring (MRM) was used at unit resolution. Collision-induced dissociation was performed using argon (5.0; Linde, Germany) at 0.8 mTorr. Data from MRM analysis were evaluated by MS Data Review (version 6.9.2, Varian, USA).

MRM transitions and the related parameters for analytes and their deuterated isotopes are summarized in ESM4_Table 1.

ESM4_Table 1 Retention times (tR) and MRM parameters for the determination of trimethylsilyl derivatives of oxyChOLs by GC-MS/MS. Time segments resulted in scan times <2 sec. m/z, mass-to-charge ratio; CE, collision energy

Analyte	t_R	m/z precursor	m/z product	CE (eV)
7β- HO-ChOL-d6	26.64	462	233	20.0
7β- HO-ChOL	26.73	456	233	20.0
5,6β-epoxy-ChOL-d6	27.15	480	390	7.5
5,6β-epoxy-ChOL	27.24	474	384	7.5
5,6α-epoxy-ChOL-d6	27.37	480	390	7.5
5,6α-epoxy-ChOL	27.45	474	384	7.5

Calibration and validation

Linear relationship of concentrations and peak area ratios of non-deuterated and deuterated analytes as well as homogeneity of variances of calibration points was verified using synthesized deuterated and commercially available unlabeled reference compounds and was statistically verified at $P < 0.05$. To cover a wide range of ratios of areas observed during measurements, several linear calibration ranges had to be applied per analyte to cover the whole range. Every calibration exhibited equidistant calibration points and was performed three times independently, starting from weighing of compounds.

Calibration points for 5,6α-epoxy-ChOL (50 - 625 pg *on column*) 5,6β-epoxy-ChOL (300 - 1300 pg *on column*), and 7β-HO-ChOL (0.18 - 13.5 pg *on column*) were prepared the same way as tissue samples.

Validation of the analytical method was performed by characterizing accuracy, precision, limit of quantification (LOQ) and limit of detection (LOD) and summarized in ESM5_Table 2).

Accuracy and precision were determined by replicate analysis of spiked and non-spiked samples containing known low, middle and high amounts of the oxyChOLs by the method described above.

LOQ was set as the smallest amount of the respective oxyChOL that could be quantified; i.e. the lowest concentration used for calibration.

LOD was determined in tissue samples using the respective deuterated standards assuming minimum signal-to-noise ratio (S/N) of 2.

ESM4_Table 2 Accuracy, precision, LOQ and LOD of oxyChOL determination in breast adipose tissue. Accuracies and precision are expressed as mean ± SD of three independent determinations for each concentration

	Accuracy (%)	Precision (%)	LOQ (ng/g tissue)	LOD (ng/g tissue)
5,6α-epoxy-ChOL	99.2 ± 1.5	6.2 ± 2.9	12.50	3.89
5,6β-epoxy-ChOL	99.4 ± 9.0	4.6 ± 1.5	66.09	1.99
7β-HO-ChOL	106.2 ± 10.3	6.0 ± 3.3	1.80	0.13

References

Pemp D, Kleider C, Schmalbach K, Hauptstein R, Geppert LN, Köllmann C, Ickstadt K, Eckert P, Neshkova I, Jakubietz, R, Esch HL, Lehmann L (2019) Qualitative and quantitative differences in estrogen biotransformation in human breast glandular and adipose tissues: implications for studies using mammary biospecimens. Arch Toxicol 93:2823-2833. https://doi.org/10.1007/s00204-019-02564-w
