# Supplementary material for: Influence of breast cancer risk factors on proliferation and DNA damage in human breast glandular tissues: role of intracellular estrogen levels, oxidative stress and estrogen biotransformation
Source: Arch Toxicol. 2021 Dec 18;96(2):673–87. doi: 10.1007/s00204-021-03198-7 (PMC8837527; doi:10.1007/s00204-021-03198-7)
Supplement: Supplementary file 5 — Supplementary file5 (PDF 785 KB) [file 204_2021_3198_MOESM5_ESM.pdf]

Archives of Toxicology

Influence of breast cancer risk factors on proliferation and DNA damage in human breast glandular tissues: role of intracellular estrogen levels, oxidative stress and estrogen biotransformation

Juliane Wunder, Daniela Pemp, Alexander Cecil, Maryam Mahdiani, René Hauptstein, Katja Schmalbach, Leo N. Geppert, Katja Ickstadt, Harald L. Esch, Thomas Dandekar, Leane Lehmann*

***Corresponding author:** Prof. Dr. Leane Lehmann, Chair of Food Chemistry, University of Würzburg, Am Hubland, D-97074 Würzburg, Germany. Phone: +49 931 318-5481. Email: leane.lehmann@uni-wuerzburg.de.

Online Resource 5 Tissue levels (part A) as well as composition of the principal components of oxycholesterols and values of principal component 1 (part B) formed by oxidative stress in 41 human breast adipose tissue derived from women without breast cancer used in multiple linear regression models.

Part A Tissue levels of 5,6α-epoxy-cholesterol (5,6α-epoxy-ChOL), 5,6β-epoxy-ChOL and 7β-hydroxy-ChOL (7β-HO-ChOL), determined by GC-MS/MS as described in Online Resource 4.

	Levels (ng/g)		
**No.**	5,6α-epoxy-ChOL	5,6β-epoxy-ChOL	7β-HO-ChOL
1	85.88	269.81	45.61
2	16.88	74.29	2.85
3	23.80	80.61	3.47
5	82.51	624.20	6.63
6	20.53	101.41	2.99
7	16.72	66.43	2.38
8	86.37	304.74	5.82
9	17.17	90.76	3.28
10	27.87	139.11	4.71
11	110.94	263.79	37.20
12	37.49	283.26	5.47
13	40.04	184.22	9.02
14	46.97	189.13	5.87
15	76.96	159.81	18.25
16	34.56	229.69	4.29
17	39.43	186.45	4.92
18	14.22	52.40	1.81
19	13.61	41.95	0.97
20	26.55	121.60	2.22
21	49.32	195.29	3.11
22	41.35	146.97	2.01
23	45.85	164.17	26.71
24	31.10	117.58	3.84
25	40.16	161.80	3.23
27	17.07	51.74	6.66
28	29.50	147.16	2.67
29	38.65	210.63	3.54
30	31.77	100.49	3.20
31	14.65	48.28	2.56
32	42.21	180.65	3.52
33	17.92	59.25	1.29
34	24.00	81.51	2.52
35	9.87	25.70	1.26
36	49.32	166.00	3.50
37	18.22	43.66	5.75
38	29.47	160.86	2.64
39	36.82	149.90	4.11
40	22.97	132.31	4.05
41	19.00	40.29	1.58
42	34.81	161.23	3.23
43	16.61	68.13	10.60

Part B Composition of principal components and values of principle component 1 calculated in R calculated with the levels of 5,6α-epoxy-ChOL, 5,6β-epoxy-ChOL and 7β-HO-ChOL in human adipose tissues. Percentage of variation, which is explained by the PCs, is given and eigenvectors of prominent variables influencing the PCs are indicated with bold characters.

*used in multiple linear regression models describing levels of transcripts indicating ESR activation and proliferation, levels of transcripts encoding enzymes in biotransformation of E2/E1, indicating (oxidative) cellular stress and on levels of calculated fluxes to E1/E2-DNA adducts. Criteria for choosing PCs are presented in Online Resource 7.

Variable	Eigenvectors		
	**PCOXY1***	**PCOXY2**	**PCOXY3**
5,6α-epoxyChOL	**- 0.7**	<0.1	**0.8**
5,6β-epoxyChOL	**- 0.6**	**0.7**	- 0.5
7β-HO-ChOL	**- 0.5**	**- 0.8**	- 0.4
% of variation explained	73	23	4

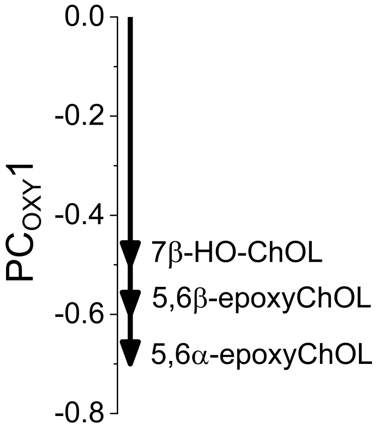

No.	PCOXY1	No.	PCOXY1
1	- 4.25	23	- 1.49
2	1.14	24	0.46
3	0.88	25	0.00
5	- 3.83	27	1.04
6	0.89	28	0.41
7	1.22	29	- 0.23
8	- 2.21	30	0.56
9	1.02	31	1.36
10	0.39	32	- 0.17
11	- 4.45	33	1.28
12	- 0.69	34	0.92
13	- 0.44	35	1.69
14	- 0.49	36	- 0.30
15	- 1.88	37	1.10
16	- 0.26	38	0.34
17	- 0.20	39	0.11
18	1.39	40	0.60
19	1.51	41	1.33
20	0.65	42	0.15
21	- 0.43	43	0.75
22	0.11		
