# Supplementary material for: Influence of breast cancer risk factors on proliferation and DNA damage in human breast glandular tissues: role of intracellular estrogen levels, oxidative stress and estrogen biotransformation
Source: Arch Toxicol. 2021 Dec 18;96(2):673–87. doi: 10.1007/s00204-021-03198-7 (PMC8837527; doi:10.1007/s00204-021-03198-7)
Supplement: Supplementary file 6 — Supplementary file6 (PDF 163 KB) [file 204_2021_3198_MOESM6_ESM.pdf]

Archives of Toxicology

Influence of breast cancer risk factors on proliferation and DNA damage in human breast glandular tissues: role of intracellular estrogen levels, oxidative stress and estrogen biotransformation

Juliane Wunder, Daniela Pemp, Alexander Cecil, Maryam Mahdiani, René Hauptstein, Katja Schmalbach, Leo N. Geppert, Katja Ickstadt, Harald L. Esch, Thomas Dandekar, Leane Lehmann*

***Corresponding author:** Prof. Dr. Leane Lehmann, Chair of Food Chemistry, University of Würzburg, Am Hubland, D-97074 Würzburg, Germany. Phone: +49 931 318-5481. Email: leane.lehmann@uni-wuerzburg.de.

Online Resource 6 Metabolic network fluxes used either for validation of the metabolic network or for multiple linear regression models.

Fluxes to 2-methoxy-estrone (2-MeO-E1), 4-methoxy-estrone (4-MeO-E1), 2-methoxy-17β-estradiol (2-MeO-E2), 4-methoxy-17β-estradiol (4-MeO-E2), the sum of the fluxes to estrone glucuronide (E1-G) and to the DNA adducts of E2 (E2-DNA) and E1 (E1-DNA) were calculated by the metabolic network model using data derived from breast glandular tissues of 44 women without breast cancer. Fluxes < 0.0001 were set as 0.0000.

Fluxes to methoxylated estrogens and E1-G were used for validation, fluxes to DNA adducts of E2 and E1 were used in multiple linear regression models

*, not used in forward selected multiple linear regression models, because of missing lack of data regarding specific explanatory variables.

No.	Metabolic network fluxes to						
	2-MeO-E1	4-MeO-E1	2-MeO-E2	4-MeO-E2	E1-G	E2-DNA	E1-DNA
1	0.0021	0.0017	0.0019	0.0015	0.057	0.0150	0.0160
2	0.0039	0.0029	0.0021	0.0021	0.137	0.0050	0.0136
3	0.0021	0.0015	0.0014	0.0014	0.084	0.0058	0.0098
4*	0.0135	0.0082	0.0029	0.0028	0.463	0.0198	0.0408
5	0.0024	0.0024	0.0024	0.0024	0.004	0.0000	0.0008
6	0.0024	0.0020	0.0020	0.0020	0.056	0.0006	0.0062
7	0.0015	0.0015	0.0015	0.0015	0.034	0.0042	0.0068
8	0.0029	0.0024	0.0024	0.0024	0.063	0.0140	0.0202
9	0.0101	0.0099	0.0095	0.0095	0.026	0.0100	0.0124
10	0.0046	0.0038	0.0043	0.0036	0.110	0.0152	0.0162
11	0.0088	0.0057	0.0062	0.0039	0.277	0.0264	0.0316
12	0.0038	0.0026	0.0041	0.0026	0.127	0.0218	0.0212
13	0.0045	0.0045	0.0045	0.0045	0.008	0.0008	0.0032
14	0.0050	0.0050	0.0050	0.0050	0.000	0.0002	0.0010
15	0.0068	0.0050	0.0071	0.0050	0.184	0.0108	0.0110
16	0.0030	0.0030	0.0030	0.0030	0.034	0.0044	0.0136
17	0.0041	0.0029	0.0031	0.0022	0.146	0.0190	0.0220
18	0.0027	0.0027	0.0027	0.0027	0.004	0.0000	0.0000
19	0.0150	0.0146	0.0143	0.0143	0.022	0.0030	0.0088
20	0.0019	0.0019	0.0019	0.0019	0.042	0.0032	0.0074
21	0.0053	0.0043	0.0054	0.0043	0.124	0.0242	0.0242
22	0.0044	0.0044	0.0044	0.0044	0.078	0.0144	0.0182
23	0.0025	0.0025	0.0025	0.0025	0.000	0.0000	0.0000
24	0.0017	0.0015	0.0015	0.0015	0.062	0.0128	0.0150
25	0.0048	0.0034	0.0061	0.0040	0.149	0.0256	0.0236
26*	0.0033	0.0033	0.0033	0.0033	0.002	0.0032	0.0084
27	0.0040	0.0039	0.0039	0.0039	0.056	0.0018	0.0068
28	0.0052	0.0035	0.0029	0.0022	0.157	0.0170	0.0218
29	0.0047	0.0047	0.0047	0.0047	0.029	0.0164	0.0198
30	0.0053	0.0037	0.0032	0.0025	0.157	0.0112	0.0156
31	0.0059	0.0042	0.0055	0.0038	0.182	0.0238	0.0252
32	0.0023	0.0023	0.0023	0.0023	0.035	0.0034	0.0072
33	0.0021	0.0021	0.0021	0.0021	0.024	0.0080	0.0090
34	0.0018	0.0015	0.0015	0.0015	0.038	0.0034	0.0070
35	0.0028	0.0028	0.0028	0.0028	0.030	0.0028	0.0094
36	0.0016	0.0016	0.0016	0.0016	0.017	0.0044	0.0070
37	0.0026	0.0026	0.0026	0.0026	0.021	0.0064	0.0072
38	0.0055	0.0048	0.0047	0.0042	0.108	0.0148	0.0168
39	0.0059	0.0049	0.0057	0.0047	0.125	0.0230	0.0236
40	0.0052	0.0040	0.0050	0.0037	0.149	0.0014	0.0018
41	0.0048	0.0048	0.0048	0.0048	0.024	0.0126	0.0122
42	0.0062	0.0054	0.0061	0.0053	0.099	0.0220	0.0226
43	0.0040	0.0040	0.0040	0.0040	0.009	0.0022	0.0034
44*	0.0138	0.0138	0.0138	0.0138	0.000	0.0054	0.0060
