# Supplementary material for: Influence of breast cancer risk factors on proliferation and DNA damage in human breast glandular tissues: role of intracellular estrogen levels, oxidative stress and estrogen biotransformation
Source: Arch Toxicol. 2021 Dec 18;96(2):673–87. doi: 10.1007/s00204-021-03198-7 (PMC8837527; doi:10.1007/s00204-021-03198-7)
Supplement: Supplementary file 7 — Supplementary file7 (PDF 1315 KB) [file 204_2021_3198_MOESM7_ESM.pdf]

Archives of Toxicology

Influence of breast cancer risk factors on proliferation and DNA damage in human breast glandular tissues: role of intracellular estrogen levels, oxidative stress and estrogen biotransformation

Juliane Wunder, Daniela Pemp, Alexander Cecil, Maryam Mahdiani, René Hauptstein, Katja Schmalbach, Leo N. Geppert, Katja Ickstadt, Harald L. Esch, Thomas Dandekar, Leane Lehmann*

***Corresponding author:** Prof. Dr. Leane Lehmann, Chair of Food Chemistry, University of Würzburg, Am Hubland, D-97074 Würzburg, Germany. Phone: +49 931 318-5481. Email: leane.lehmann@uni-wuerzburg.de.

Online Resource 7 Setup and outcome of multiple linear regression models using stepwise forward selection.

To test the association of every possible explanatory variable (exVAR) with the dependent variable, the variable explaining the dependent variable best is chosen by an automatic procedure. Subsequently, all possible exVAR are added one after another to the first one, ultimately choosing the one improving the model most, applying the Akaike information criterion. This is repeated until the model cannot be further improved by adding exVARs. Thus, every exVAR selected into the model contributes to modeling the dependent variable. Significance of the association is expressed by P values and magnitude of impact is expressed by coefficients of regression.

To identify variables influencing levels of transcripts indicating ESR activation (*AREG, GATA3, TFF1, PGR, TGFB1, WNT4*) or proliferation (*CCND1, CDKN1A, CDKN1B*), levels of calculated fluxes to 17β-estradiol (E2)-DNA adducts and estrone (E1)-DNA adducts calculated in the metabolic network model, levels of transcripts encoding enzymes in biotransformation of E2 and E1 (*CYP1A1, CYP1B1, NQO1, GSTT1, GSTP1, COMT, STS, SULT1A3/4, SULT1A2*) and markers for (oxidative) cellular stress (*GCLC;* oxy-cholesterols, oxyChOLs) in human breast tissue, up to 17 explanatory variables (exVARs, continuous as well as binary and categorical) were tested as possible exVARs. ExVARs tested were: Age (continuous), menopausal status (categorical, reference group premenopausal, PreMP, n = 22), and further groups perimenopausal (PeriMP, n = 12), and postmenopausal (PostMP, n = 7), lobule type (categorical, reference group lobule type 2/3, Lob2/3, n = 10), and further groups lobule type 1 nulliparous (Lob1np, n = 16), and lobule type 1 parous (Lob1p, n = 15, Pemp et al. 2020). To reflect continuous influence of age as well as abrupt influence of menopausal status on dependent variables, both the potential exVARs age and menopause, were included into the models. Further exVARs tested were oil% in glandular tissue (continuous), body mass index (BMI, continuous), smoking habits (binary, reference group non-smoker, (n = 31, smoker n = 10), intake of estrogen active drugs (EAD, categorial, reference group no intake of EAD, n = 33) and further groups intake of ethinyl estradiol (EE, n = 6), and intake of E2-releasing drugs (ERD, n = 2), intake of dietary supplements containing phytoestrogens (IPE, binary, reference group no intake of IPE, n = 35, intake n = 6), levels of transcripts of *GCLC* (continuous) as marker of oxidative stress glandular tissue, levels of transcripts of estrogen receptor (*ESR*)*1* and *ESR2* (both continuous) and levels of 2-methoxy (2MeO)-E1 < and >LOD (binary, reference group <LOD; Pemp et al. 2020). Since a multitude

of Spearman correlations between exVARs was observed (data not shown), to minimize collinearity, levels of estrogens in glandular tissue and oxyChOLs caused by oxidative stress in adipose tissue were grouped in principal components (PCs) PC_E1 and $PC_{OXY}1$; respectively. Likewise, levels of transcripts indicating ESR activation were grouped in principal components $PC_{EA}1$ and $PC_{EA}2$.

Number of PCs, which were calculated for specific groups of exVARs, were chosen using the scree plots according to the elbow criteria and all principal components considered explained at least two third of the variation of the original variables. The composition of principal components considered in the multiple linear regression models as well as the percentage of variation, explained by the respective PCs and eigenvectors of prominent variables influencing the PCs are presented in Online Resource 2, Online Resource 3, Online Resource 5.

If in the computed model observations with Cook's Distance >1 appeared, they were removed and the model was computed anew. This process was repeated until no conspicuous observations (CO) remained. To achieve normal distribution, all dependent variables except for levels of transcripts of *CCND1, CDKN1B, UGT1A9, CYP1B1, GST T1, GST P1, NQO1*, for levels of calculated fluxes to E2- and E1-DNA adducts and values of $PC_{OXY}1$ were logarithmized. Data distributions were evaluated in Quantile-Quantile plots with simulated confidence bands. Constant standard deviations of the errors were evaluated using scale-location plots. To check the model assumption of independent identically distributed errors, the residual vs. fitted values plot was used. For every final model, adjusted coefficient of determination (R^2), the numbers of CO removed, the number of observations contributing to the final model (n, maximum 41, two specimens without information on EAD and one specimen without information on oxyChOLs, and the ratio of observations per exVAR (O/exVAR) is given. Low R^2 of the respective linear regression models indicate lack of variables contributing to the dependent variable and/or large variations in the data. For every exVAR selected, the regression coefficients (which represent the mean changes in the dependent variables for one unit of change in the respective exVAR while holding other predictors in the models constant), their confidence interval (CI), as well as the P values were given.

Spearman's rank correlation analysis was performed to identify collinearity between exVAR which might hinder each others selection and/or influence each others P values within the models. Correlations with P values < 0.001 were listed below the table of each model with the respective correlation coefficient (R) and P value. If such exVARs were selected for the final model, a comment addressing possible consequences were added. Associations of exVARs selected and exhibiting P values ≤ 0.20 are interpreted in the last column of each table.

The order of dependent variables corresponds to their appearance in Fig. 2, Fig. 3, Fig. 5 and Fig. 6.

+/-, positive/negative association; ↑, increase; ↓, decrease; n.a., not applicable; black colored exVAR selected, P values < 0.05; blue colored exVAR selected, P values between ≥ 0.05-0.10; light-blue colored exVAR selected, P values between ≥ 0.10-0.20; grey colored exVAR selected, P values > 0.20.

AREG

CO = 0; n = 41; O/exVAR = 5.1; $R^2 = 0.53$

exVAR	Regression Coefficient	Influence via PC	CI 2.5%	CI 97.5%	*P* Value	Hypothesized sign of regression coefficient or influence via PC on levels of the dependent variable
Age[1]	- 0.0194	n.a.	- 0.0534	+ 0.0146	0.2539	+/-, if effect in addition to changes in endogenous estrogen levels
PeriMP[2]	+ 0.3337	n.a.	- 0.4065	+ 1.0739	0.3653	+/-, if effect in addition to decrease in endogenous estrogen levels
PostMP[3]	- 0.5663	n.a.	- 1.5723	+ 0.4397	0.2600	+/-, if effect in addition to decrease in endogenous estrogen levels
Lob1np		n.a.				+/-, if effect in addition to changes in endogenous estrogen levels
Lob1p		n.a.				+/-, if effect in addition to decrease in endogenous estrogen levels
Oil%		n.a.				+/-, if effect in addition to increase in endogenous estrogen levels
BMI		n.a.				+/-, if effect in addition to increase in endogenous estrogen levels
Smoking		n.a.				+/-, if effect in addition to changes in endogenous estrogen levels
EAD:EE	+ 0.4775	n.a.	- 0.1695	+ 1.1246	0.1426	+, if intake contributes to ESR1 activation
EAD:ERD	+ 0.6909	n.a.	- 0.3174	+ 1.6992	0.1724	+, if effect in addition to increase in endogenous estrogen levels
GCLC[4]		n.a.				+/-, if short-term marker for cellular (oxidative) stress affect ESR1 activation
						+, if co-regulation
$PC_{OXY}1$		n.a.				+/-, if long-term oxidative stress affects ESR1 activation
PC_E1	+ 0.1852	n.a.	+ 0.0332	+ 0.3371	0.0185	+, if ESR1 activation is affected quantitatively by endogenous estrogen levels
		+E2				
		+E1				
		+E1S				
ESR1[1,2,3]		n.a.				+, if transcript levels reflect protein levels, and if protein levels limit ESR1 response
ESR2[4]	- 10.6706	n.a.	- 22.0406	+ 0.6995	0.0649	+, if transcript levels reflect protein activity and ESR2 and ESR1 act agonistically
		n.a.				-, if transcript levels reflect protein activity and ESR2 and ESR1 act antagonistically
IPE	+ 0.6568	n.a.	+ 0.0677	+ 1.2459	0.0300	+/-, if intake affects ESR1 activation

Comment:

Because of selection of the exVAR Age into the model maybe no selection of the exVAR *ESR1*[1].

Because of selection of the exVAR *ESR2* into the model maybe no selection of the exVAR *GCLC*[4].

Note:

[1], Correlation between Age and *ESR1*: $r = 0.54$, $P = 2.50 \times 10^{-4}$.

[2], Significant difference between values of *ESR1* in women PeriMP (higher) and PreMP (lower): $P < 0.001$ (8.73×10^{-5}).

[3], Significant difference between values of *ESR1* in women postMP (higher) and PreMP (lower): $P < 0.001$ (9.33×10^{-4}).

[4], Correlation of *GCLC* and *ESR2*: $r = 0.62$, $P = 1.81 \times 10^{-5}$.

GATA3

CO = 1; n = 40; O/exVAR = 40.0; $R^2 = 0.23$

exVAR	Regression Coefficient	Influence via PC	CI 2.5%	CI 97.5%	*P* Value	Hypothesized sign of regression coefficient or influence via PC on levels of the dependent variable
Age		n.a.				+/-, if effect in addition to changes in endogenous estrogen levels
PeriMP		n.a.				+/-, if effect in addition to decrease in endogenous estrogen levels
PostMP		n.a.				+/-, if effect in addition to decrease in endogenous estrogen levels
Lob1np		n.a.				+/-, if effect in addition to changes in endogenous estrogen levels
Lob1p		n.a.				+/-, if effect in addition to decrease in endogenous estrogen levels
Oil%		n.a.				+/-, if effect in addition to increase in endogenous estrogen levels
BMI		n.a.				+/-, if effect in addition to increase in endogenous estrogen levels
Smoking		n.a.				+/-, if effect in addition to changes in endogenous estrogen levels
EAD:EE		n.a.				+, if intake contributes to ESR1 activation
EAD:ERD		n.a.				+, if effect in addition to increase in endogenous estrogen levels
GCLC		n.a.				+/-, if short-term marker for cellular (oxidative) stress affect ESR1 activation<br>+, if co-regulation
$PC_{OXY}1$		n.a.				+/-, if long-term oxidative stress affects ESR1 activation
PC_E1	+ 0.2141	+E2<br>+E1<br>+E1S	+ 0.0916	+ 0.3366	0.0011	+, if ESR1 activation is affected quantitatively by endogenous estrogen levels
ESR1		n.a.				+, if transcript levels reflect protein levels, and if protein levels limit ESR1 response
ESR2		n.a.				+, if transcript levels reflect protein activity and ESR2 and ESR1 act agonistically<br>-, if transcript levels reflect protein activity and ESR2 and ESR1 act antagonistically
IPE		n.a.				+/-, if intake affects ESR1 activation

TFF1

CO = 0; n = 41; O/exVAR = 4.6; $R^2 = 0.48$

exVAR	Regression Coefficient		Influence via PC	CI 2.5%		CI 97.5%		*P* Value	Hypothesized sign of regression coefficient or influence via PC on levels of the dependent variable
Age			n.a.						+/-, if effect in addition to changes in endogenous estrogen levels
PeriMP[1]	+	1.2880	n.a.	+	0.0693	+	2.5068	0.0390	+/-, if effect in addition to decrease in endogenous estrogen levels
PostMP[2]	-	0.6952	n.a.	-	2.2644	+	0.8739	0.3732	+/-, if effect in addition to decrease in endogenous estrogen levels
Lob1np	+	0.6396	n.a.	-	0.8778	+	2.1569	0.3965	+/-, if effect in addition to changes in endogenous estrogen levels
Lob1p	-	0.5395	n.a.	-	1.9820	+	0.9030	0.4514	+/-, if effect in addition to decrease in endogenous estrogen levels
Oil%			n.a.						+/-, if effect in addition to increase in endogenous estrogen levels
BMI			n.a.						+/-, if effect in addition to increase in endogenous estrogen levels
Smoking			n.a.						+/-, if effect in addition to changes in endogenous estrogen levels
EAD:EE	+	0.8260	n.a.	-	0.6780	+	2.3301	0.2713	+, if intake contributes to ESR1 activation
EAD:ERD	-	1.9861	n.a.	-	4.3550	+	0.3828	0.0973	+, if effect in addition to increase in endogenous estrogen levels
GCLC[3]			n.a.						+/-, if short-term marker for cellular (oxidative) stress affect ESR1 activation<br>+, if co-regulation
$PC_{OXY}1$			n.a.						+/-, if long-term oxidative stress affects ESR1 activation
PC_E1	+	0.5808	+E2<br>+E1<br>+E1S	+	0.2208	+	0.9408	0.0025	+, if ESR1 activation is affected quantitatively by endogenous estrogen levels
ESR1[1,2]			n.a.						+, if transcript levels reflect protein levels, and if protein levels limit ESR1 response
ESR2[3]	-	39.2214	n.a.	-	65.1782	-	13.2646	0.0043	+, if transcript levels reflect protein activity and ESR2 and ESR1 act agonistically<br>-, if transcript levels reflect protein activity and ESR2 and ESR1 act antagonistically
IPE	+	1.7741	n.a.	+	0.3901	+	3.1581	0.0137	+/-, if intake affects ESR1 activation

Comment:

Because of selection of the exVAR PeriMP into the model maybe no selection of the exVAR *ESR1*[2].
Because of selection of the exVAR *ESR2* into the model maybe no selection of the exVAR *GCLC*[4].

Note:

[1], Significant difference between values of *ESR1* in women PeriMP (higher) and PreMP (lower): $P < 0.001$ (8.73×10^{-5}).
[2], Significant difference between values of *ESR1* in women PostMP (higher) and PreMP (lower): $P < 0.001$ (9.33×10^{-4}).
[3], Correlation between *GCLC* and *ESR2*: $r = 0.62$, $P = 1.81 \times 10^{-5}$.

PGR

CO = 0; n = 41; O/exVAR = 5.1; R^2 = 0.47

exVAR	Regression Coefficient	Influence via PC	CI 2.5%	CI 97.5%	*P* Value	Hypothesized sign of regression coefficient or influence via PC on levels of the dependent variable
Age[1]		n.a.				+/-, if effect in addition to changes in endogenous estrogen levels
PeriMP[2]	+ 0.2944	n.a.	- 0.1277	+ 0.7165	0.1651	+/-, if effect in addition to decrease in endogenous estrogen levels
PostMP[3]	- 0.4521	n.a.	- 1.0633	+ 0.1590	0.1416	+/-, if effect in addition to decrease in endogenous estrogen levels
Lob1np	+ 0.0541	n.a.	- 0.4146	+ 0.5228	0.8156	+/-, if effect in addition to changes in endogenous estrogen levels
Lob1p	- 0.5658	n.a.	- 1.1038	- 0.0279	0.0399	+/-, if effect in addition to decrease in endogenous estrogen levels
Oil%	+ 0.0101	n.a.	- 0.0035	+ 0.0237	0.1405	+/-, if effect in addition to increase in endogenous estrogen levels
BMI	+ 0.0606	n.a.	+ 0.0171	+ 0.1041	0.0079	+/-, if effect in addition to increase in endogenous estrogen levels
Smoking		n.a.				+/-, if effect in addition to changes in endogenous estrogen levels
EAD:EE		n.a.				+, if intake contributes to ESR1 activation
EAD:ERD		n.a.				+, if effect in addition to increase in endogenous estrogen levels
GCLC		n.a.				+/-, if short-term marker for cellular (oxidative) stress affect ESR1 activation<br>+, if co-regulation
$PC_{OXY}1$		n.a.				+/-, if long-term oxidative stress affects ESR1 activation
$PC_{E}1$	+ 0.1582	 +E2<br>+E1<br>+E1S	+ 0.0224	+ 0.2940	0.0238	+, if ESR1 activation is affected quantitatively by endogenous estrogen levels
ESR1[1,2,3]	+ 0.0910	n.a.	+ 0.0087	+ 0.1733	0.0314	+, if transcript levels reflect protein levels, and if protein levels limit ESR1 response
ESR2		n.a.				+, if transcript levels reflect protein activity and ESR2 and ESR1 act agonistically<br>-, if transcript levels reflect protein activity and ESR2 and ESR1 act antagonistically
IPE		n.a.				+/-, if intake affects ESR1 activation

Comment:

Because of selection of the exVAR *ESR1* into the model maybe no selection of the exVAR Age[1].

Note:

[1], Correlation between Age and *ESR1*: $r = 0.54$, $P = 2.50 \times 10^{-4}$.

[2], Significant difference between values of *ESR1* in women PeriMP (higher) and PreMP (lower): $P < 0.001$ (8.73×10^{-5}).

[3], Significant difference between values of *ESR1* in women PostMP (higher) and PreMP (lower): $P < 0.001$ (9.33×10^{-4}).

TGFB1

CO = 0; n = 41; O/exVAR = 6.8; R^2 = 0.39

exVAR	Regression Coefficient		Influence via PC	CI 2.5%		97.5%		*P* Value	Hypothesized sign of regression coefficient or influence via PC on levels of the dependent variable
Age			n.a.						+/-, if effect in addition to changes in endogenous estrogen levels
PeriMP			n.a.						+/-, if effect in addition to decrease in endogenous estrogen levels
PostMP			n.a.						+/-, if effect in addition to decrease in endogenous estrogen levels
Lob1np	+	0.4186	n.a.	+	0.0813	+	0.7559	0.0165	+/-, if effect in addition to changes in endogenous estrogen levels
Lob1p	+	0.1774	n.a.	-	0.1484	+	0.5032	0.2763	+/-, if effect in addition to decrease in endogenous estrogen levels
Oil%	+	0.0065	n.a.	-	0.0033	+	0.0162	0.1859	+/-, if effect in addition to increase in endogenous estrogen levels
BMI	+	0.0359	n.a.	+	0.0040	+	0.0678	0.0286	+/-, if effect in addition to increase in endogenous estrogen levels
Smoking			n.a.						+/-, if effect in addition to changes in endogenous estrogen levels
EAD:EE			n.a.						+, if intake contributes to ESR1 activation
EAD:ERD			n.a.						+, if effect in addition to increase in endogenous estrogen levels
GCLC[1]			n.a.						+/-, if short-term marker for cellular (oxidative) stress affect ESR1 activation<br>+, if co-regulation
$PC_{OXY}1$			n.a.						+/-, if long-term oxidative stress affects ESR1 activation
PC_E1			n.a.						+, if ESR1 activation is affected quantitatively by endogenous estrogen levels
ESR1			n.a.						+, if transcript levels reflect protein levels, and if protein levels limit ESR1 response
ESR2[1]	+	9.7854	n.a.	+	2.6591	+	16.9117	0.0086	+, if transcript levels reflect protein activity and ESR2 and ESR1 act agonistically<br>-, if transcript levels reflect protein activity and ESR2 and ESR1 act antagonistically
IPE	+	0.4028	n.a.	+	0.0346	+	0.7709	0.0330	+/-, if intake affects ESR1 activation

Comment:
Because of selection of the exVAR *ESR2* into the model maybe no selection of the exVAR *GCLC*.

Note:
[1], Correlation between *GCLC* and *ESR2*: $r = 0.62$, $P = 1.81 \times 10^{-5}$.

WNT4

CO = 2; n = 39; O/exVAR = 7.8; $R^2 = 0.52$

exVAR	Regression Coefficient	Influence via PC	CI 2.5%	CI 97.5%	*P* Value	Hypothesized sign of regression coefficient or influence via PC on levels of the dependent variable
Age		n.a.				+/-, if effect in addition to changes in endogenous estrogen levels
PeriMP[1]	- 0.7318	n.a.	- 1.1173	- 0.3463	0.0005	+/-, if effect in addition to decrease in endogenous estrogen levels
PostMP[2]	- 0.9889	n.a.	- 1.4239	- 0.5538	< 0.0001	+/-, if effect in addition to decrease in endogenous estrogen levels
Lob1np		n.a.				+/-, if effect in addition to changes in endogenous estrogen levels
Lob1p		n.a.				+/-, if effect in addition to decrease in endogenous estrogen levels
Oil%	+ 0.0088	n.a.	- 0.0031	+ 0.0207	0.1428	+/-, if effect in addition to increase in endogenous estrogen levels
BMI		n.a.				+/-, if effect in addition to increase in endogenous estrogen levels
Smoking	- 0.6064	n.a.	- 1.0802	- 0.1326	0.0137	+/-, if effect in addition to changes in endogenous estrogen levels
EAD:EE	+ 0.5246	n.a.	- 0.0379	+ 1.0871	0.0665	+, if intake contributes to ESR1 activation
EAD:ERD		n.a.				+, if effect in addition to increase in endogenous estrogen levels
GCLC		n.a.				+/-, if short-term marker for cellular (oxidative) stress affect ESR1 activation
						+, if co-regulation
$PC_{OXY}1$		n.a.				+/-, if long-term oxidative stress affects ESR1 activation
$PC_{E}1$		n.a.				+, if ESR1 activation is affected quantitatively by endogenous estrogen levels
ESR1[1,2]		n.a.				+, if transcript levels reflect protein levels, and if protein levels limit ESR1 response
ESR2		n.a.				+, if transcript levels reflect protein activity and ESR2 and ESR1 act agonistically
						-, if transcript levels reflect protein activity and ESR2 and ESR1 act antagonistically
IPE		n.a.				+/-, if intake affects ESR1 activation

Comment:

Because of selection of the exVAR PeriMP into the model maybe no selection of the exVAR *ESR1*[2].

Because of selection of the exVAR PostMP into the model maybe no selection of the exVAR *ESR1*[3].

Note:

[1], Significant difference between values of *ESR1* in women PeriMP (higher) and PreMP (lower): $P < 0.001$ (8.73×10^{-5}).

[2], Significant difference between values of *ESR1* in women PostMP (higher) and PreMP (lower): $P < 0.001$ (9.33×10^{-4}).

CCND1

CO = 0; n = 41; O/exVAR = 10.3; $R^2 = 0.24$

exVAR	Regression Coefficient	Influence via PC	CI 2.5%	CI 97.5%	*P* Value	Hypothesized sign of regression coefficient or influence via PC on levels of the dependent variable
Age[1]	- 0.0705	n.a.	- 0.1205	- 0.0205	0.0070	+/-, if effect in addition to ESR1 activation
PeriMP		n.a.				+/-, if effect in addition to ESR1 activation
PostMP[2]		n.a.				+/-, if effect in addition to ESR1 activation
Lob1np		n.a.				+/-, if effect in addition to ESR1 activation
Lob1p		n.a.				+/-, if effect in addition to ESR1 activation
Oil%		n.a.				+/-, if effect in addition to ESR1 activation
BMI		n.a.				+/-, if effect in addition to ESR1 activation
Smoking		n.a.				+, Ah receptor activation
EAD:EE		n.a.				+, if intake contributes to *CCND1* expression
EAD:ERD		n.a.				+, if effect in addition to ESR1 activation
GCLC		n.a.				+/-, if short-term marker for cellular (oxidative) stress affect *CCND1* expression<br>+, if co-regulation
$PC_{OXY}1$		n.a.				+/-, if long-term oxidative stress affects *CCND1* expression
GATA3		n.a.				+, if *CCND1* expression is induced by ESR1 activation in addition to $PC_{EA}1$
TGFB1		n.a.				-, if levels reflect levels of active protein
$PC_{EA}1$[2]	+ 0.3198	*+AREG*<br>*+PGR*<br>*+TFF1*	- 0.0678	+ 0.7074	0.1030	+, if *CCND1* expression is induced by ESR1 activation
$PC_{EA}2$[1]	- 0.5677	*-WNT4*	- 1.1206	- 0.0148	0.0445	-, if levels reflect levels of active protein
IPE	+ 1.3843	n.a.	- 0.2753	+ 3.0438	0.0994	+/-, if intake affects *CCND1* expression

Comment:

Because of the strong correlation between exVARs Age and $PC_{EA}2$, *P* values of both are possibly too high[1].

Because of selection of the exVAR $PC_{EA}1$ into the model maybe no selection of the exVAR PostMP[2].

Note:

[1], Correlation between Age and $PC_{EA}2$: $r = -0.52$, $P = 4.50 \times 10^{-4}$.

[2], Significant difference between values of $PC_{EA}1$ in women PostMP (lower) and PreMP (higher): $P < 0.001$ (5.13×10^{-6}).

CDKN1A

CO = 1; n = 40; O/exVAR = 10.0; R^2 = 0.60

exVAR	Regression Coefficient		Influence via PC	CI 2.5%		CI 97.5%		*P* Value	Hypothesized sign of regression coefficient or influence via PC on levels of the dependent variable
Age[1]	+	0.0119	n.a.	-	0.0006	+	0.0244	0.0606	+/-, if effect in addition to ESR1 activation
PeriMP			n.a.						+/-, if effect in addition to ESR1 activation
PostMP			n.a.						+/-, if effect in addition to ESR1 activation
Lob1np			n.a.						+/-, if effect in addition to ESR1 activation
Lob1p			n.a.						+/-, if effect in addition to ESR1 activation
Oil%			n.a.						+/-, if effect in addition to ESR1 activation
BMI			n.a.						+/-, if effect in addition to ESR1 activation
Smoking			n.a.						+, Ah receptor activation
EAD:EE			n.a.						+, if intake contributes to *CDKN1A* expression
EAD:ERD			n.a.						+/-, if effect in addition to ESR1 activation
GCLC	+	1.0894	n.a.	+	0.6331	+	1.5458	< 0.0001	+/-, if short-term marker for cellular (oxidative) stress affect *CDKN1A* expression<br>+, if co-regulation
$PC_{OXY}1$			n.a.						+/-, if long-term oxidative stress affects *CDKN1A* expression
GATA3	+	0.0774	n.a.	+	0.0251	+	0.1298	0.0049	+, if *CDKN1A* expression is induced by ESR1 activation in addition to $PC_{EA}1$
TGFB1	+	0.2515	n.a.	-	0.0023	+	0.5052	0.0520	-, if levels reflect levels of active protein
$PC_{EA}1$			n.a.						+, if *CDKN1A* expression is induced by ESR1 activation
$PC_{EA}2$[1]			n.a.						-, if levels reflect levels of active protein
IPE			n.a.						+/-, if intake affects *CDKN1A* expression

Comment:
Because of selection of the exVAR Age into the model maybe no selection of the exVAR $PC_{EA}2$[1].

Note:
[1], Correlation between Age and PC_{EA} 2: $r = -0.52$, $P = 4.50 \times 10^{-4}$.

CDKN1B

CO = 0; n = 41; O/exVAR = 6.8; $R^2 = 0.54$

exVAR	Regression Coefficient	Influence via PC	CI 2.5%	CI 97.5%	*P* Value	Hypothesized sign of regression coefficient or influence via PC on levels of the dependent variable
Age		n.a.				+/-, if effect in addition to ESR1 activation
PeriMP		n.a.				+/-, if effect in addition to ESR1 activation
PostMP[1]		n.a.				+/-, if effect in addition to ESR1 activation
Lob1np		n.a.				+/-, if effect in addition to ESR1 activation
Lob1p		n.a.				+/-, if effect in addition to ESR1 activation
Oil%	+ 0.0265	n.a.	- 0.0050	+ 0.0580	0.0969	+/-, if effect in addition to ESR1 activation
BMI	- 0.1001	n.a.	- 0.1980	- 0.0023	0.0452	+/-, if effect in addition to ESR1 activation
Smoking		n.a.				+, Ah receptor activation
EAD:EE		n.a.				+, if intake contributes to *CDKN1B* expression
EAD:ERD		n.a.				+/-, if effect in addition to ESR1 activation
GCLC	+ 0.7882	n.a.	- 0.3758	+ 1.9523	0.1778	+/-, if short-term marker for cellular (oxidative) stress affect *CDKN1B* expression<br>+, if co-regulation
$PC_{OXY}1$		n.a.				+/-, if long-term oxidative stress affects *CDKN1B* expression
GATA3		n.a.				+, if *CDKN1B* expression is induced by ESR1 activation in addition to $PC_{EA}1$
TGFB1	+ 1.3273	n.a.	+ 0.6112	+ 2.0433	0.0006	-, if levels reflect levels of active protein
$PC_{EA}1$[1]	+ 0.2962		+ 0.0299	+ 0.5625	0.0303	+, if *CDKN1B* expression is induced by ESR1 activation
		+*AREG*<br>+*PGR*<br>+*TFF1*				
$PC_{EA}2$		n.a.				-, if levels reflect levels of active protein
IPE	+ 0.8610	n.a.	- 0.2661	+ 1.9880	0.1298	+/-, if intake affects *CDKN1B* expression

Comment:

Because of selection of the exVAR $PC_{EA}1$ into the model maybe no selection of the exVAR PostMP[2].

Note:

[1], Significant difference between values of $PC_{EA}1$ in women PostMP (lower) and PreMP (higher): $P < 0.001$ (5.13×10^{-6}).

UGT1A9

CO = 2; n = 39; O/exVAR = 7.8; $R^2 = 0.36$

**exVAR**	**Regression Coefficient**	**Influence via PC**	**CI 2.5%**	**CI 97.5%**	***P* Value**	**Hypothesized sign of regression coefficient or influence via PC on levels of the dependent variable**
Age		n.a.				+/-, if effect in addition to changes in endogenous estrogen levels
PeriMP		n.a.				+/-, if effect in addition to decrease in endogenous estrogen levels
PostMP		n.a.				+/-, if effect in addition to decrease in endogenous estrogen levels
Lob1np		n.a.				+/-, if effect in addition to changes in endogenous estrogen levels
Lob1p		n.a.				+/-, if effect in addition to decrease in endogenous estrogen levels
Oil%		n.a.				+/-, if effect in addition to increase in endogenous estrogen levels
BMI	- 0.2277	n.a.	- 0.3744	- 0.0810	0.0034	+/-, if effect in addition to increase in endogenous estrogen levels
Smoking		n.a.				+/-, if effect in addition to changes in endogenous estrogen levels
						+, Ah receptor activation
EAD:EE	- 1.6594	n.a.	- 3.4104	+ 0.0915	0.0625	+, if intake contributes to regulation of transcript levels
EAD:ERD	+ 1.3468	n.a.	- 1.3346	+ 4.0281	0.3143	+, if effect in addition to increase in endogenous estrogen levels
GCLC	- 1.2275	n.a.	- 2.9200	+ 0.4650	0.1496	+, if expression is induced by short-term marker for cellular (oxidative) stress
						-, if *UGT1A9* levels reflect enzyme activities
$PC_{OXY}1$		n.a.				+, if expression is induced by long-term oxidative stress
2-MeOE1 >LOD		n.a.				+/-, if levels of 2-MeO-E1 are indicative for increase in oxidative metabolism and expression is affected by cellular (oxidative) stress
						-, if *UGT1A9* levels reflect enzyme activities
$PC_{E}1$		n.a.				+, if expression is induced by endogenous estrogen levels
IPE	- 2.0000	n.a.	- 3.7278	- 0.2723	0.0246	+/-, if intake contributes to regulation of transcript levels

UGT1A10

CO = 0; n = 41; O/exVAR = 8.2; $R^2 = 0.31$

exVAR	Regression Coefficient	Influence via PC	CI 2.5%	CI 97.5%	*P* Value	Hypothesized sign of regression coefficient or influence via PC on levels of the dependent variable
Age		n.a.				+/-, if effect in addition to changes in endogenous estrogen levels
PeriMP	+ 0.0002	n.a.	+ < 0.0001	+ 0.0003	0.0447	+/-, if effect in addition to changes in endogenous estrogen levels
PostMP	+ 0.0002	n.a.	+ < 0.0001	+ 0.0004	0.0102	+/-, if effect in addition to changes in endogenous estrogen levels
Lob1np		n.a.				+/-, if effect in addition to changes in endogenous estrogen levels
Lob1p		n.a.				+/-, if effect in addition to changes in endogenous estrogen levels
Oil%		n.a.				+/-, if effect in addition to changes in endogenous estrogen levels
BMI		n.a.				+/-, if effect in addition to changes in endogenous estrogen levels
Smoking	+ 0.0001	n.a.	+ < 0.0001	+ < 0.0001	0.0493	+/-, if effect in addition to changes in endogenous estrogen levels<br>+, Ah receptor activation
EAD:EE		n.a.				+, if intake contributes to regulation of transcript levels
EAD:ERD		n.a.				+, if effect in addition to increase in endogenous estrogen levels
GCLC	- 0.0002	n.a.	- 0.0004	+ < 0.0001	0.0872	+, if expression is induced by short-term marker for cellular (oxidative) stress<br>-, if *UGT1A10* levels reflect enzyme activities
$PC_{OXY}1$		n.a.				+, if expression is induced by long-term oxidative stress
2-MeOE1 >LOD		n.a.				+/-, if levels of 2-MeO-E1 are indicative for increase in oxidative metabolism and expression is affected by cellular (oxidative) stress<br>-, if *UGT1A10* levels reflect enzyme activities
$PC_E 1$		n.a.				+, if expression is induced by endogenous estrogen levels
IPE	- 0.0002	n.a.	- 0.0004	- > 0.0001	0.0094	+/-, if intake contributes to regulation of transcript levels

CYP1B1

CO = 0; n = 41; O/exVAR = 20.5; $R^2 = 0.18$

**exVAR**	**Regression Coefficient**	**Influence via PC**	**CI 2.5%**	**CI 97.5%**	***P* Value**	**Hypothesized sign of regression coefficient or influence via PC on levels of the dependent variable**
Age		n.a.				+/-, if effect in addition to changes in endogenous estrogen levels
PeriMP		n.a.				+/-, if effect in addition to changes in endogenous estrogen levels
PostMP		n.a.				+/-, if effect in addition to changes in endogenous estrogen levels
Lob1np		n.a.				+/-, if effect in addition to changes in endogenous estrogen levels
Lob1p		n.a.				+/-, if effect in addition to changes in endogenous estrogen levels
Oil%		n.a.				+/-, if effect in addition to changes in endogenous estrogen levels
BMI	+ 0.0487	n.a.	- 0.0013	+ 0.0987	0.0559	+/-, if effect in addition to changes in endogenous estrogen levels
Smoking	+ 0.7693	n.a.	+ 0.2863	+ 1.2523	0.0026	+/-, if effect in addition to changes in endogenous estrogen levels<br>+, Ah receptor activation
EAD:EE		n.a.				+, if intake contributes to regulation of transcript levels
EAD:ERD		n.a.				+, if effect in addition to increase in endogenous estrogen levels
GCLC		n.a.				+, if expression is induced by short-term marker for cellular (oxidative) stress
$PC_{OXY}1$		n.a.				+, if expression is induced by long-term oxidative stress
2-MeOE1 >LOD		n.a.				-, if levels of 2-MeO-E1 are indicative for increase in oxidative metabolism and expression is decreased by cellular (oxidative) stress
PC_E1		n.a.				+, if expression is induced by endogenous estrogen levels
IPE		n.a.				+, if intake contributes to regulation of transcript levels

CYP1A1

CO = 1; n = 40; O/exVAR = 13.3; $R^2 = 0.51$

exVAR	Regression Coefficient	Influence via PC	CI 2.5%	CI 97.5%	*P* Value	Hypothesized sign of regression coefficient or influence via PC on levels of the dependent variable
Age		n.a.				+/-, if effect in addition to changes in endogenous estrogen levels
PeriMP		n.a.				+/-, if effect in addition to changes in endogenous estrogen levels
PostMP		n.a.				+/-, if effect in addition to changes in endogenous estrogen levels
Lob1np		n.a.				+/-, if effect in addition to changes in endogenous estrogen levels
Lob1p		n.a.				+/-, if effect in addition to changes in endogenous estrogen levels
Oil%		n.a.				+/-, if effect in addition to changes in endogenous estrogen levels
BMI		n.a.				+/-, if effect in addition to changes in endogenous estrogen levels
Smoking	+ 3.2536	n.a.	+ 2.0134	+ 4.4937	< 0.0001	+/-, if effect in addition to changes in endogenous estrogen levels<br>+, Ah receptor activation
EAD:EE		n.a.				+, if intake contributes to regulation of transcript levels
EAD:ERD		n.a.				+, if effect in addition to increase in endogenous estrogen levels
GCLC	+ 1.4744	n.a.	- 0.0273	+ 2.9761	0.0541	+, if expression is induced by short-term marker for cellular (oxidative) stress
$PC_{OXY}1$	+ 0.3685	-5,6α-epoxyChOL<br>-5,6β-epoxyChOL<br>-7β-HO-ChOL	+ < 0.0001	+ 0.7370	0.0500	+, if expression is induced by long-term oxidative stress
2-MeOE1 >LOD		n.a.				-, if levels of 2-MeO-E1 are indicative for increase in oxidative metabolism and expression is decreased by cellular (oxidative) stress
$PC_{E}1$		n.a.				+, if expression is induced by endogenous estrogen levels
IPE		n.a.				+, if intake contributes to regulation of transcript levels

NQO1

CO = 1; n = 40; O/exVAR = 13.3; R^2 = 0.41

**exVAR**	**Regression Coefficient**	**Influence via PC**	**CI 2.5%**	**97.5%**	***P* Value**	**Hypothesized sign of regression coefficient or influence via PC on levels of the dependent variable**
Age		n.a.				+/-, if effect in addition to changes in endogenous estrogen levels
PeriMP	+ 0.3538	n.a.	- 0.4426	+ 1.1503	0.3736	+/-, if effect in addition to changes in endogenous estrogen levels
PostMP	+ 1.6194	n.a.	+ 0.7540	+ 2.4848	0.0005	+/-, if effect in addition to changes in endogenous estrogen levels
Lob1np		n.a.				+/-, if effect in addition to changes in endogenous estrogen levels
Lob1p		n.a.				+/-, if effect in addition to changes in endogenous estrogen levels
Oil%		n.a.				+/-, if effect in addition to changes in endogenous estrogen levels
BMI		n.a.				+/-, if effect in addition to changes in endogenous estrogen levels
Smoking		n.a.				+/-, if effect in addition to changes in endogenous estrogen levels<br>+, Ah receptor activation
EAD:EE		n.a.				+, if intake contributes to regulation of transcript levels
EAD:ERD		n.a.				+, if effect in addition to increase in endogenous estrogen levels
GCLC	+ 1.8874	n.a.	+ 0.9077	+ 2.8671	0.0004	+, if expression is induced by short-term marker for cellular (oxidative) stress
$PC_{OXY}1$		n.a.				+, if expression is induced by long-term oxidative stress
2-MeOE1 >LOD		n.a.				+, if levels of 2-MeO-E1 are indicative for increase in oxidative metabolism and expression is increased by cellular (oxidative) stress
PC_E1		n.a.				+, if expression is induced by endogenous estrogen levels
IPE		n.a.				+, if intake contributes to regulation of transcript levels

GSTT1

CO = 3; n = 39; O/exVAR = 13.0; $R^2 = 0.45$

exVAR	Regression Coefficient	Influence via PC	CI 2.5%	CI 97.5%	*P* Value	Hypothesized sign of regression coefficient or influence via PC on levels of the dependent variable
Age		n.a.				+/-, if effect in addition to changes in endogenous estrogen levels
PeriMP		n.a.				+/-, if effect in addition to changes in endogenous estrogen levels
PostMP		n.a.				+/-, if effect in addition to changes in endogenous estrogen levels
Lob1np		n.a.				+/-, if effect in addition to changes in endogenous estrogen levels
Lob1p		n.a.				+/-, if effect in addition to changes in endogenous estrogen levels
Oil%	+ 0.0119	n.a.	+ 0.0028	+ 0.0210	0.0115	+/-, if effect in addition to changes in endogenous estrogen levels
BMI		n.a.				+/-, if effect in addition to changes in endogenous estrogen levels
Smoking		n.a.				+/-, if effect in addition to changes in endogenous estrogen levels
EAD:EE		n.a.				+, if intake contributes to regulation of transcript levels
EAD:ERD		n.a.				+, if effect in addition to increase in endogenous estrogen levels
GCLC	+ 0.7006	n.a.	+ 0.3793	+ 1.0220	< 0.0001	+, if expression is induced by short-term marker forcellular (oxidative) stress<br>-, if *GSTT1* levels reflect enzyme activities
$PC_{OXY}1$	- 0.0715	+5,6α-epoxyChOL<br>+5,6β-epoxyChOL<br>+7β-HO-ChOL	- 0.1483	+ 0.0054	0.0673	+, if expression is induced by long-term oxidative stress<br>-, if *GSTT1* levels reflect enzyme activities
2-MeOE1 >LOD		n.a.				+, if levels of 2-MeO-E1 are indicative for increase in oxidative metabolism and expression is increased by cellular (oxidative) stress
PC_E1		n.a.				+, if expression induced by endogenous estrogen levels
IPE		n.a.				+, if intake contributes to regulation of transcript levels

GSTP1

CO = 0; n = 41; O/exVAR = 8.2; $R^2 = 0.40$

exVAR	Regression Coefficient	Influence via PC	CI 2.5%	CI 97.5%	*P* Value	Hypothesized sign of regression coefficient or influence via PC on levels of the dependent variable
Age		n.a.				+/-, if effect in addition to changes in endogenous estrogen levels
PeriMP		n.a.				+/-, if effect in addition to changes in endogenous estrogen levels
PostMP		n.a.				+/-, if effect in addition to changes in endogenous estrogen levels
Lob1np	- 0.6450	n.a.	- 5.2159	+ 3.9258	0.7762	+/-, if effect in addition to changes in endogenous estrogen levels
Lob1p	+ 4.2589	n.a.	+ 0.0492	+ 8.4685	0.0475	+/-, if effect in addition to changes in endogenous estrogen levels
Oil%		n.a.				+/-, if effect in addition to changes in endogenous estrogen levels
BMI		n.a.				+/-, if effect in addition to changes in endogenous estrogen levels
Smoking	+ 7.1792	n.a.	+ 3.1719	+ 11.1865	0.0009	+/-, if effect in addition to changes in endogenous estrogen levels
EAD:EE		n.a.				+, if intake contributes to regulation of transcript levels
EAD:ERD		n.a.				+, if effect in addition to increase in endogenous estrogen levels
GCLC		n.a.				+, if expression is induced by short-term marker for cellular (oxidative) stress<br>-, if *GSTP1* levels reflect enzyme activities
$PC_{OXY}1$	+ 1.1801		+ 0.0281	+ 2.3322	0.0450	+, if expression is induced by long-term oxidative stress<br>-, if *GSTP1* levels reflect enzyme activities
		-5,6α-epoxyChOL<br>-5,6β-epoxyChOL<br>-7β-HO-ChOL				
2-MeOE1 >LOD		n.a.				+, if levels of 2-MeO-E1 are indicative for increase in oxidative metabolism and expression is increased by cellular (oxidative) stress
PC_E1		n.a.				+, if expression induced by endogenous estrogen levels
IPE	- 5.8445	n.a.	- 10.8027	- 0.8864	0.0222	+, if intake contributes to regulation of transcript levels

COMT

CO = 1; n = 40; O/exVAR = 20.0; $R^2 = 0.22$

exVAR	Regression Coefficient		Influence via PC	CI 2.5%		97.5%		*P* Value	Hypothesized sign of regression coefficient or influence via PC on levels of the dependent variable
Age			n.a.						+/-, if effect in addition to changes in endogenous estrogen levels
PeriMP			n.a.						+/-, if effect in addition to changes in endogenous estrogen levels
PostMP			n.a.						+/-, if effect in addition to changes in endogenous estrogen levels
Lob1np			n.a.						+/-, if effect in addition to changes in endogenous estrogen levels
Lob1p			n.a.						+/-, if effect in addition to changes in endogenous estrogen levels
Oil%			n.a.						+/-, if effect in addition to changes in endogenous estrogen levels
BMI	+	0.0275	n.a.	-	0.0081	+	0.0631	0.1260	+/-, if effect in addition to changes in endogenous estrogen levels
Smoking			n.a.						+/-, if effect in addition to changes in endogenous estrogen levels
EAD:EE			n.a.						+, if intake contributes to regulation of transcript levels
EAD:ERD			n.a.						+, if effect in addition to increase in endogenous estrogen levels
GCLC	+	0.6754	n.a.	+	0.2584	+	1.0925	0.0023	+, if expression is induced by short-term marker for cellular (oxidative) stress<br>-, if *COMT* levels reflect enzyme activities
$PC_{OXY}1$			n.a.						+, if expression is induced by long-term oxidative stress
2-MeOE1 >LOD			n.a.						+/-, if levels of 2-MeO-E1 are indicative for increase in oxidative metabolism and expression is affected by cellular (oxidative) stress<br>+, if *COMT* levels reflect enzyme activities
PC_E1			n.a.						+, if expression induced by endogenous estrogen levels
IPE			n.a.						+, if intake contributes to regulation of transcript levels

STS

CO = 1; n = 40; O/exVAR = 10.0; $R^2 = 0.26$

exVAR	Regression Coefficient		Influence via PC	CI 2.5%		CI 97.5%		*P* Value	Hypothesized sign of regression coefficient or influence via PC on levels of the dependent variable
Age	-	0.0284	n.a.	-	0.0550	-	0.0019	0.0366	+/-, if effect in addition to changes in endogenous estrogen levels
PeriMP	+	0.8048	n.a.	+	0.1951	+	1.4145	0.0112	+/-, if effect in addition to changes in endogenous estrogen levels
PostMP	+	0.4680	n.a.	-	0.3384	+	1.2744	0.2467	+/-, if effect in addition to changes in endogenous estrogen levels
Lob1np			n.a.						+/-, if effect in addition to changes in endogenous estrogen levels
Lob1p			n.a.						+/-, if effect in addition to changes in endogenous estrogen levels
Oil%			n.a.						+/-, if effect in addition to changes in endogenous estrogen levels
BMI			n.a.						+/-, if effect in addition to changes in endogenous estrogen levels
Smoking			n.a.						+/-, if effect in addition to changes in endogenous estrogen levels
EAD:EE			n.a.						+, if intake contributes to regulation of transcript levels
EAD:ERD			n.a.						+, if effect in addition to increase in endogenous estrogen levels
GCLC	+	0.4851	n.a.	-	0.0337	+	1.0040	0.0660	+, if expression is induced by short-term marker for cellular (oxidative) stress
$PC_{OXY}1$			n.a.						+/-, if levels of 2-MeO-E1 are indicative for increase in oxidative metabolism and expression is affected by cellular (oxidative) stress
2-MeOE1 >LOD			n.a.						+/-, if levels of 2-MeO-E1 are indicative for increase in oxidative metabolism and expression is affected by cellular (oxidative) stress
PC_E1			n.a.						+, if expression induced by endogenous estrogen levels
IPE			n.a.						+, if intake contributes to regulation of transcript levels

SULT1A1

CO = 0; n = 41; O/exVAR = 8.2; $R^2 = 0.43$

exVAR	Regression Coefficient		Influence via PC	CI 2.5%		CI 97.5%		*P* Value	Hypothesized sign of regression coefficient or influence via PC on levels of the dependent variable
Age			n.a.						+/-, if effect in addition to changes in endogenous estrogen levels
PeriMP	+	0.4957	n.a.	+	0.1499	+	0.8415	0.0063	+/-, if effect in addition to changes in endogenous estrogen levels
PostMP	+	0.1646	n.a.	-	0.2363	+	0.5655	0.4103	+/-, if effect in addition to changes in endogenous estrogen levels
Lob1np			n.a.						+/-, if effect in addition to changes in endogenous estrogen levels
Lob1p			n.a.						+/-, if effect in addition to changes in endogenous estrogen levels
Oil%			n.a.						+/-, if effect in addition to changes in endogenous estrogen levels
BMI	-	0.0467	n.a.	-	0.0809	-	0.0126	0.0087	+/-, if effect in addition to changes in endogenous estrogen levels
Smoking			n.a.						+/-, if effect in addition to changes in endogenous estrogen levels
EAD:EE			n.a.						+, if intake contributes to regulation of transcript levels
EAD:ERD			n.a.						+, if effect in addition to increase in endogenous estrogen levels
GCLC	+	0.4158	n.a.	-	0.0264	+	0.8581	0.0645	+, if expression is induced by short-term marker for cellular (oxidative) stress<br>-, if *SULT1A1* levels reflect enzyme activities
$PC_{OXY}1$	-	0.1590	+5,6α-epoxyChOL<br>+5,6β-epoxyChOL<br>+7β-HO-ChOL	-	0.2585	-	0.0594	0.0026	+, if expression is induced by long-term oxidative stress
2-MeOE1 >LOD			n.a.						+/-, if levels of 2-MeO-E1 are indicative for increase in oxidative metabolism and expression is affected by cellular (oxidative) stress<br>-, if *SULT1A1* levels reflect enzyme activities
PC_E1			n.a.						+, if expression induced by endogenous estrogen levels
IPE			n.a.						+, if intake contributes to regulation of transcript levels

SULT1A2

CO = 0; n = 41; O/exVAR = 6.8; R^2 = 0.40

exVAR	Regression Coefficient	Influence via PC	CI 2.5%	CI 97.5%	*P* Value	Hypothesized sign of regression coefficient or influence via PC on levels of the dependent variable
Age		n.a.				+/-, if effect in addition to changes in endogenous estrogen levels
PeriMP	+ 0.4868	n.a.	+ 0.0086	+ 0.9650	0.0462	+/-, if effect in addition to changes in endogenous estrogen levels
						+/-, if effect in addition to changes in endogenous estrogen levels
PostMP	+ 0.1954	n.a.	- 0.4388	+ 0.8296	0.5354	+/-, if effect in addition to changes in endogenous estrogen levels
Lob1np	+ 0.2453	n.a.	- 0.2712	+ 0.7619	0.3412	+/-, if effect in addition to changes in endogenous estrogen levels
Lob1p	+ 0.8793	n.a.	+ 0.2999	+ 1.4586	0.0040	+/-, if effect in addition to changes in endogenous estrogen levels
						+/-, if effect in addition to changes in endogenous estrogen levels
Oil%		n.a.				+/-, if effect in addition to changes in endogenous estrogen levels
BMI		n.a.				+/-, if effect in addition to changes in endogenous estrogen levels
Smoking	+ 0.5667	n.a.	+ 0.0736	+ 1.0596	0.0256	+/-, if effect in addition to changes in endogenous estrogen levels
EAD:EE		n.a.				+, if intake contributes to regulation of transcript levels
EAD:ERD		n.a.				+, if effect in addition to increase in endogenous estrogen levels
GCLC		n.a.				+, if expression is induced by short-term marker for cellular (oxidative) stress
						-, if *SULT1A2* levels reflect enzyme activities
$PC_{OXY}1$	- 0.2064		- 0.3398	- 0.0730	0.0034	+, if expression is induced by long-term oxidative stress
		+5,6α-epoxyChOL				
		+5,6β-epoxyChOL				
		+7β-HO-ChOL				
2-MeOE1 >LOD		n.a.				+/-, if levels of 2-MeO-E1 are indicative for increase in oxidative metabolism and expression is affected by cellular (oxidative) stress
						-, if *SULT1A2* levels reflect enzyme activities
PC_E1		n.a.				+, if expression induced by endogenous estrogen levels
IPE		n.a.				+, if intake contributes to regulation of transcript levels

SULT1A3/4

CO = 0; n = 41; O/exVAR = 41.0; $R^2 = 0.03$

exVAR	Regression Coefficient	Influence via PC	CI 2.5%	CI 97.5%	*P* Value	Hypothesized sign of regression coefficient or influence via PC on levels of the dependent variable
Age		n.a.				+/-, if effect in addition to changes in endogenous estrogen levels
PeriMP		n.a.				+/-, if effect in addition to decrease in endogenous estrogen levels
PostMP		n.a.				+/-, if effect in addition to decrease in endogenous estrogen levels
Lob1np		n.a.				+/-, if effect in addition to changes in endogenous estrogen levels
Lob1p		n.a.				+/-, if effect in addition to decrease in endogenous estrogen levels
Oil%		n.a.				+/-, if effect in addition to increase in endogenous estrogen levels
BMI		n.a.				+/-, if effect in addition to increase in endogenous estrogen levels
Smoking		n.a.				+/-, if effect in addition to changes in endogenous estrogen levels
EAD:EE		n.a.				+, if intake contributes to regulation of transcript levels
EAD:ERD		n.a.				+, if effect in addition to increase in endogenous estrogen levels
GCLC	+ 0.4064	n.a.	- 0.1690	+ 0.9819	0.1610	+, if expression is induced by short-term marker for cellular (oxidative) stress<br>-, if *SULT1A3/4* levels reflect enzyme activities
$PC_{OXY}1$		n.a.				+, if expression is induced by long-term oxidative stress
2-MeOE1 >LOD		n.a.				+/-, if levels of 2-MeO-E1 are indicative for increase in oxidative metabolism and expression is affected by cellular (oxidative) stress<br>-, if *SULT1A3/4* levels reflect enzyme activities
$PC_{E}1$		n.a.				+, if expression induced by endogenous estrogen levels
IPE		n.a.				+/-, if intake contributes to regulation of transcript levels

SULT1E1

CO = 0; n = 41; O/exVAR = 20.5; R^2 = 0.29

exVAR	Regression Coefficient	Influence via PC	CI 2.5%	CI 97.5%	*P* Value	Hypothesized sign of regression coefficient or influence via PC on levels of the dependent variable
Age	+ 0.0749	n.a.	+ 0.0381	+ 0.1116	0.0002	+/-, if effect in addition to changes in endogenous estrogen levels
PeriMP		n.a.				+/-, if effect in addition to changes in endogenous estrogen levels
PostMP		n.a.				+/-, if effect in addition to changes in endogenous estrogen levels
Lob1np		n.a.				+/-, if effect in addition to changes in endogenous estrogen levels
Lob1p		n.a.				+/-, if effect in addition to changes in endogenous estrogen levels
Oil%		n.a.				+/-, if effect in addition to changes in endogenous estrogen levels
BMI		n.a.				+/-, if effect in addition to changes in endogenous estrogen levels
Smoking		n.a.				+/-, if effect in addition to changes in endogenous estrogen levels
EAD:EE		n.a.				+, if intake contributes to regulation of transcript levels
EAD:ERD		n.a.				+, if effect in addition to increase in endogenous estrogen levels
GCLC	- 0.9847	n.a.	- 2.2540	+ 0.2846	0.1246	+, if expression is induced by short-term marker for cellular (oxidative) stress<br>-, if *SULT1E1* levels reflect enzyme activities
$PC_{OXY}1$		n.a.				+, if expression is induced by long-term oxidative stress
2-MeOE1 >LOD		n.a.				+/-, if levels of 2-MeO-E1 are indicative for increase in oxidative metabolism and expression is affected by cellular (oxidative) stress<br>-, if *SULT1E1* levels reflect enzyme activities
PC_E1		n.a.				+, if expression induced by endogenous estrogen levels
IPE		n.a.				+, if intake contributes to regulation of transcript levels

GCLC

CO = 0; n = 41; O/exVAR = 5.1; R^2 = 0.34

exVAR	Regression Coefficient	Influence via PC	CI 2.5%	CI 97.5%	*P* Value	Hypothesized sign of regression coefficient or influence via PC on levels of the dependent variable
Age		n.a.				+/-, if effect in addition to changes in endogenous estrogen levels
PeriMP	+ 0.2672	n.a.	- 0.0029	+ 0.5373	0.0524	+/-, if effect in addition to changes in endogenous estrogen levels
PostMP	- 0.2581	n.a.	- 0.6056	+ 0.0895	0.1402	+/-, if effect in addition to changes in endogenous estrogen levels
Lob1np	+ 0.1381	n.a.	- 0.1815	+ 0.4577	0.3854	+/-, if effect in addition to changes in endogenous estrogen levels
Lob1p	+ 0.3824	n.a.	+ 0.0645	+ 0.7002	0.0199	+/-, if effect in addition to changes in endogenous estrogen levels
Oil%	+ 0.0064	n.a.	- 0.0015	+ 0.0144	0.1104	+/-, if effect in addition to changes in endogenous estrogen levels
BMI		n.a.				+/-, if effect in addition to changes in endogenous estrogen levels
Smoking		n.a.				+/-, if effect in addition to changes in endogenous estrogen levels
EAD:EE	+ 0.4109	n.a.	+ 0.0605	+ 0.7613	0.0230	+, if intake contributes to regulation of transcript levels
EAD:ERD	- 0.1652	n.a.	- 0.6783	+ 0.3479	0.5166	+, if effect in addition to increase in endogenous estrogen levels
$PC_{OXY}1$		n.a.				+/-, if levels of 2-MeO-E1 are indicative for increase in oxidative metabolism and expression is affected by cellular (oxidative) stress
2-MeOE1 >LOD		n.a.				+, if levels of 2-MeO-E1 are indicative for increase in oxidative metabolism and expression is increased by cellular (oxidative) stress
PC_E1		n.a.				+, if expression induced by endogenous estrogen levels
IPE	+ 0.2877	n.a.	- 0.0329	+ 0.6083	0.0769	+, if intake contributes to regulation of transcript levels

$PC_{OXY}1$

CO = 3; n = 38; O/exVAR = 7.6; R^2 = 0.42

exVAR	Regression Coefficient	Influence on oxyChOLs via PCs and exVars	CI 2.5%	CI 97.5%	*P* Value	Hypothesized sign of influence on oxyChOLs via PC and exVARs
Age		n.a.				+, if contributing to cellular oxidative stress in breast tissue
PeriMP		n.a.				+, if contributing to cellular oxidative stress in breast tissue
PostMP		n.a.				+, if contributing to cellular oxidative stress in breast tissue
Lob1np		n.a.				+, if contributing to cellular oxidative stress in breast tissue
Lob1p		n.a.				+, if contributing to cellular oxidative stress in breast tissue
Oil%	+ 0.0337	negative	+ 0.0091	+ 0.0582	0.0087	+, if contributing to cellular oxidative stress in breast tissue
BMI		n.a.				+, if contributing to cellular oxidative stress in breast tissue
Smoking		n.a.				+, if contributing to cellular oxidative stress in breast tissue
EAD:EE	+ 0.2799	negative	- 0.5376	+ 1.0974	0.4906	+, if intake contributes to cellular oxidative stress
EAD:ERD	+ 1.7789	negative	+ 0.4385	+ 3.1192	0.0109	+, if effect in addition to increase in endogenous estrogen levels
GCLC		n.a.				+, if equivalent to marker for long-term cellular oxidative stress
NQO1		n.a.				+, if equivalent to marker for long-term cellular oxidative stress
2-MeOE1 >LOD[1]	- 1.4926	positive	- 2.7095	- 0.2757	0.0178	+, if levels of 2-MeO-E1 are indicative for increased cellular oxidative stress
PC_E1[1]	- 0.2351	+E2<br>+E1<br>+E1S	- 0.4937	+ 0.0236	0.0734	+, if contributing to cellular oxidative stress in breast tissue
IPE		n.a.				+, if intake contributes to cellular oxidative stress

Comment:

P values of exVARs PC_E1 and 2-MeOE1 >LOD possibly too high, because of strong correlation between them, respectively[1].

Note:

[1], Significant difference of values of PC_E1 in women 2-MeOE1 >LOD (2-MeOE1 >LOD (higher) and <LOD (lower): $P < 0.001$ (3.02×10^{-4}).

E2 DNA adduct flux (identification of reactions within the metabolic network model)

CO = 2; n = 39; O/exVAR = 4.9; R^2 = 0.94

exVAR	Regression Coefficient	Influence via PC	CI 2.5%	CI 97.5%	*P* Value	Hypothesized sign of regression coefficient or influence via PC on levels of the dependent variable
COMT		n.a.				-
CYP1A1		n.a.				+
CYP1B1	+ 0.0034	n.a.	+ 0.0022	+ 0.00464	<0.0001	+
GSTP1	- 0.0005	n.a.	- > 0.0001	- 0.0004	<0.0001	-
GSTT1		n.a.				-
NQO1	- 0.0012	n.a.	- 0.0018	- > 0.0001	<0.0001	-
STS	- 0.0004	n.a.	- > 0.0001	+ < 0.0001	0.0884	+
SULT1A1		n.a.				-
SULT1A2	- 0.0360	n.a.	- 0.0641	- 0.0078	0.0141	-
SULT1A3/4		n.a.				-
SULT1E1	+ 0.898	n.a.	+ 0.2426	+ 1.5533	0.0089	-
UGT1A9[1]		n.a.				-
UGT1A10[1]		n.a.				-
E2[2]	+ <0.0001	n.a.	+ < 0.0001	+ < 0.0001	<0.0001	+
E1[2]	+ <0.0001	n.a.	+ < 0.0001	+ < 0.0001	<0.0001	+

Comment:
Because of the strong correlation between exVARs E1 and E2, *P* value of each is possibly too high[2].

Note:
[1], Correlation between UGT1A9 and UGT1A10: $r = 0.78$, $P = 1.72 \times 10^{-9}$.
[2], Correlation between E1 and E2: $r = 0.63$, $P = 7.82 \times 10^{-6}$.

E1 DNA adduct flux (identification of reactions within the metabolic network model)

CO = 1; n = 40; O/exVAR = 4.0; R^2 = 0.93

exVAR	Regression Coefficient		Influence via PC	CI 2.5%		CI 97.5%		*P* Value	Hypothesized sign of regression coefficient or influence via PC on levels of the dependent variable
COMT	-	0.0026	n.a.	-	0.0043	-	> 0.0001	0.0053	-
CYP1A1			n.a.						+
CYP1B1	+	0.0046	n.a.	+	0.0032	+	0.0060	< 0.0001	+
GSTP1	-	> 0.0001	n.a.	-	> 0.0001	-	> 0.0001	< 0.0001	-
GSTT1			n.a.						-
NQO1	-	0.0011	n.a.	-	0.0017	-	> 0.0001	0.0018	-
STS	+	0.0006	n.a.	-	0.0002	+	0.0015	0.1511	+
SULT1A1	-	0.0308	n.a.	-	0.0513	-	0.0103	0.0047	-
SULT1A2	+	0.0988	n.a.	-	0.0054	+	0.0203	0.0621	-
SULT1A3/4	+	0.0062	n.a.	-	> 0.0001	+	0.0131	0.0787	-
SULT1E1			n.a.						-
UGT1A9[1]			n.a.						-
UGT1A10[1]			n.a.						-
E2[2]	+	< 0.0001	n.a.	+	< 0.0001	+	< 0.0001	< 0.0001	+
E1[2]	+	< 0.0001	n.a.	-	> 0.0001	+	< 0.0001	0.2036	+

Comment:

Because of the strong correlation between exVARs E1 and E2, *P* value of each is possibly too high[2].

Note:

[1], Correlation of UGT1A9 and UGT1A10: $r = 0.78$, $P = 1.72 \times 10^{-9}$.

[2], Correlation of E1 and E2: $r = 0.63$, $P = 7.82 \times 10^{-6}$.

E2 DNA adduct flux (identification of influence of BCRFs)

CO = 0; n = 41; O/exVAR = 8.2; R^2 = 0.28

exVAR	Regression Coefficient	Influence via PC	CI 2.5%	CI 97.5%	*P* Value	Hypothesized sign of regression coefficient or influence via PC on levels of the dependent variable
Age		n.a.				
PeriMP		n.a.				
PostMP		n.a.				
Lob1np	- 0.0058	n.a.	- 0.0116	+ < 0.0001	0.0501	
Lob1p	- 0.0099	n.a.	- 0.0158	- 0.0041	0.0014	
Oil%	+ 0.0001	n.a.	- > 0.0001	+ 0.0003	0.1293	
BMI	+ 0.0008	n.a.	+ 0.0002	+ 0.0013	0.0078	All:
Smoking		n.a.				+/-, if effect on levels of estrogens or transcripts encoding enzymes
EAD:EE		n.a.				involved key reactions of the metabolic network model (i.e. *CYP1B1*,
EAD:ERD		n.a.				*GSTP1, NQO1 SULT1A2, SULT1E1)*
GCLC		n.a.				
$PC_{OXY}1$	- 0.0013		- 0.0028	+ 0.0002	0.0971	
		+5,6α-epoxyChOL				
		+5,6β-epoxyChOL				
		+7β-HO-ChOL				
IPE		n.a.				

E1 DNA adduct flux (identification of influence of BCRFs)
CO = 0; n = 41; O/exVAR = 4.6; R^2 = 0.37

exVAR	Regression Coefficient	Influence via PC	CI 2.5%	CI 97.5%	*P* Value	Hypothesized sign of regression coefficient or influence via PC on levels of the dependent variable
Age		n.a.				
PeriMP	- 0.0023	n.a.	- 0.0081	+ 0.0035	0.4256	
PostMP	+ 0.0067	n.a.	- 0.0141	+ 0.0007	0.0729	
Lob1np	- 0.0032	n.a.	- 0.0094	+ 0.0029	0.2930	
Lob1p	- 0.0069	n.a.	- 0.0135	- 0.0002	0.0425	
Oil%	+ 0.0001	n.a.	- > 0.0001	+ 0.0003	0.0916	
BMI	+ 0.0006	n.a.	+ < 0.0001	+ 0.0011	0.0429	All:
Smoking		n.a.				+/-, if effect on levels of estrogens or transcripts encoding enzymes
EAD:EE	- 0.0025	n.a.	- 0.0010	+ 0.0052	0.5122	involved key reactions of the metabolic network model (i.e. *COMT*,
EAD:ERD	+ 0.0088	n.a.	- 0.0017	+ 0.0194	0.0985	*CYP1B1*, *GSTP1, NQO1, SULT1A2, SULT1A1, SULT1E1*).
GCLC		n.a.				
$PC_{OXY}1$	- 0.0011	+5,6α-epoxyChOL<br>+5,6β-epoxyChOL<br>+7β-HO-ChOL	- 0.0026	+ 0.0004	0.1487	
IPE		n.a.				

References

Pemp D, Geppert LN., Wigmann, C, Kleider C, Hauptstein R, Schmalbach K, Ickstadt K, Esch HL, Lehmann L (2020). Influence of breast cancer risk factors and intramammary biotransformation on estrogen homeostasis in the human breast. Arch Toxicol 94:3013–3025. https://doi.org/10.1007/s00204-020-02807-1
