# Supplementary material for: Influence of breast cancer risk factors on proliferation and DNA damage in human breast glandular tissues: role of intracellular estrogen levels, oxidative stress and estrogen biotransformation
Source: Arch Toxicol. 2021 Dec 18;96(2):673–87. doi: 10.1007/s00204-021-03198-7 (PMC8837527; doi:10.1007/s00204-021-03198-7)
Supplement: Supplementary file 8 — Supplementary file8 (PDF 112 KB) [file 204_2021_3198_MOESM8_ESM.pdf]

Archives of Toxicology

Influence of breast cancer risk factors on proliferation and DNA damage in human breast glandular tissues: role of intracellular estrogen levels, oxidative stress and estrogen biotransformation

J Juliane Wunder, Daniela Pemp, Alexander Cecil, Maryam Mahdiani, René Hauptstein, Katja Schmalbach, Leo N. Geppert, Katja Ickstadt, Harald L. Esch, Thomas Dandekar, Leane Lehmann*

***Corresponding author:** Prof. Dr. Leane Lehmann, Chair of Food Chemistry, University of Würzburg, Am Hubland, D-97074 Würzburg, Germany. Phone: +49 931 318-5481. Email: leane.lehmann@uni-wuerzburg.de.

Online Ressource 8 Comparison of the fluxes to the metabolites of 17β-estradiol (E2) and estrone (E1) considered in the metabolic network model calculation. The individual fluxes of the different reaction pathways are shown as percentage of the total flux of all reactions involved in the E2/E1 metabolism. If more than two (iso)enzymes are involved, reactions are summarized according to enzyme families, E, estrogen (E2/E1); G, glucuronide; -SG, glutathione adduct; HO, hydroxy; MeO, methoxy; S, sulfate.

Reaction	(Iso) Enzyme(s)	% of total flux E2	E1
E → E-S	SULTs	1.3	1.0
E-S → E	STS	1.3	1.0
E → E	HSD17Bs	1.5	1.5
E → E-G	UGTs	4.7	4.7
E → 2-HO-E	CYP1A1	2.4	2.4
E → 2-/4-HO-E	CYP1B1	5.3	5.3
2-HO-E → 2-HO-E-S	SULTs	<0.1	<0.1
2-HO-E → 2-HO-E-G	UGTs	2.5	2.5
2-HO-E → 2-HO-3-MeO-E and 2-MeO-E	COMT	4.2	4.2
2-MeO-E → 2-MeO-E-G	UGTs	0.2	0.1
2-MeO-E → 2-HO-E	CYP1A1/ 1B1	4.1	4.1
2-HO-3-MeO-E → 2-HO-E	CYP1A1/ 1B1	4.0	4.0
2-HO-E → 2,3-E-quinone	CYP1A1/ 1B1	3.6	3.6
2-MeO-E and 2-HO-3-MeO-E → 2-MeO-E-G	UGTs	0.2	0.2
2,3-E-quinone → 2-HO-E	NQO1	1.3	1.3
2,3-E-quinone → 2-HO-E-SG	GSTs	0.6	0.6
2,3-E-quinone → E-DNA-adducts	-	1.7	1.7
4-HO-E → 4-HO-E-S	SULTs	< 0.1	<0.1
4-HO-E → 4-HO-E-G	UGTs	1.5	1.5
4-HO-E → 4-HO-3-MeO-E and 4-MeO-E	COMT	4.2	4.2
4-MeO-E → 4-MeO-E-G	UGTs	<0.1	<0.1
4-MeO-E → 4-HO-E	CYP1A1/ 1B1	4.2	4.2
4-HO-3-MeO-E → 4-HO-E	CYP1A1/ 1B1	4.2	4.2
4-HO-E → 3,4-E-quinone	CYP1A1/ 1B1	2.9	2.9
3,4-E-quinone → 4-HO-E	NQO1	1.6	1.6
3,4-E-quinone → 4-HO-E-SG	GSTs	0.3	0.3
3,4-E-quinone → E-DNA-adducts	-	0.9	0.9
